# Haplotype genomic prediction of phenotypic values based on chromosome distance and gene boundaries using low-coverage sequencing in Duroc pigs

**DOI:** 10.1186/s12711-021-00661-y

**Published:** 2021-10-07

**Authors:** Cheng Bian, Dzianis Prakapenka, Cheng Tan, Ruifei Yang, Di Zhu, Xiaoli Guo, Dewu Liu, Gengyuan Cai, Yalan Li, Zuoxiang Liang, Zhenfang Wu, Yang Da, Xiaoxiang Hu

**Affiliations:** 1 State Key Laboratory for Agrobiotechnology, College of Biological Sciences, China Agricultural University, Beijing, 100193 China; 2 Department of Animal Science, University of Minnesota, Saint Paul, MN 55108 USA; 3 College of Animal Science and National Engineering Research Center for Breeding Swine Industry, South China Agricultural University, Guangzhou, 510642 China; 4 National Engineering Research Center for Breeding Swine Industry, Wens Foodstuff Group Co., Ltd., Yunfu, 527400 China

## Abstract

**Background:**

Genomic selection using single nucleotide polymorphism (SNP) markers has been widely used for genetic improvement of livestock, but most current methods of genomic selection are based on SNP models. In this study, we investigated the prediction accuracies of haplotype models based on fixed chromosome distances and gene boundaries compared to those of SNP models for genomic prediction of phenotypic values. We also examined the reasons for the successes and failures of haplotype genomic prediction.

**Methods:**

We analyzed a swine population of 3195 Duroc boars with records on eight traits: body judging score (BJS), teat number (TN), age (AGW), loin muscle area (LMA), loin muscle depth (LMD) and back fat thickness (BF) at 100 kg live weight, and average daily gain (ADG) and feed conversion rate (FCR) from 30 to100 kg live weight. Ten-fold validation was used to evaluate the prediction accuracy of each SNP model and each multi-allelic haplotype model based on 488,124 autosomal SNPs from low-coverage sequencing. Haplotype blocks were defined using fixed chromosome distances or gene boundaries.

**Results:**

Compared to the best SNP model, the accuracy of predicting phenotypic values using a haplotype model was greater by 7.4% for BJS, 7.1% for AGW, 6.6% for ADG, 4.9% for FCR, 2.7% for LMA, 1.9% for LMD, 1.4% for BF, and 0.3% for TN. The use of gene-based haplotype blocks resulted in the best prediction accuracy for LMA, LMD, and TN. Compared to estimates of SNP additive heritability, estimates of haplotype epistasis heritability were strongly correlated with the increase in prediction accuracy by haplotype models. The increase in prediction accuracy was largest for BJS, AGW, ADG, and FCR, which also had the largest estimates of haplotype epistasis heritability, 24.4% for BJS, 14.3% for AGW, 14.5% for ADG, and 17.7% for FCR. SNP and haplotype heritability profiles across the genome identified several genes with large genetic contributions to phenotypes: *NUDT3* for LMA, LMD and BF, *VRTN* for TN, *COL5A2* for BJS, *BSND* for ADG, and *CARTPT* for FCR.

**Conclusions:**

Haplotype prediction models improved the accuracy for genomic prediction of phenotypes in Duroc pigs. For some traits, the best prediction accuracy was obtained with haplotypes defined using gene regions, which provides evidence that functional genomic information can improve the accuracy of haplotype genomic prediction for certain traits.

**Supplementary Information:**

The online version contains supplementary material available at 10.1186/s12711-021-00661-y.

## Background

Genomic prediction using single nucleotide polymorphism (SNP) markers has been widely used for livestock, but most current methods of genomic prediction use additive SNP models and only a limited number of studies have used haplotype models [1–10]. In general, these studies have achieved little to substantial improvement in prediction accuracies when using haplotype compared to SNP models. Methods used to define haplotype blocks for genomic prediction included a fixed number of SNPs per haplotype block [1, 2, 4, 9, 10], a fixed block length [8, 9], or linkage disequilibrium (LD) blocks [3, 5–7, 9]. However, none of the previous haplotype prediction models has used gene information or SNP dominance effects. A recent study using human data showed that functional genome information, including gene information, was relevant to the accuracy of haplotype genomic prediction of phenotypes, primarily as a result of haplotype epistasis, and that less accurate estimation of SNP effects by haplotype models was responsible for the failures of haplotype genomic prediction [11]. However, to date, the use of functional genomic information for haplotype genomic prediction and assessment of the successes and failures of haplotype genomic prediction have not been reported in swine.

Low-coverage sequencing (LCS) of a large number of individuals has proven to be more informative than sequencing fewer individuals at higher coverage because of the use of shared stretches of the genome across the population and haplotype diversity [12, 13]. With advances in sequencing and imputation algorithms, LCS can cover almost the whole genome and capture most of the variation in the population with high accuracy at low cost, making LCS a powerful and cost-effective genotyping tool.

In this study, we conducted an extensive evaluation of the accuracy of haplotype models for genomic prediction of phenotypic values for eight traits in Duroc pigs, using fixed chromosome distances and gene boundaries to define haplotype blocks of SNPs from LCS. For each method of haplotype block construction, one haplotype model, two SNP models, and three models that combine SNP and haplotype effects were evaluated for prediction accuracy. We also investigated the reasons for the successes and failures of the haplotype models using relative haplotype epistasis heritability and the comparison of SNP and haplotype heritability profiles [11].

## Methods

### Animals and phenotyping

Animal and phenotype data used for this study were provided by the Guangdong Wen’s Foodstuff Group (Guangdong, China). The swine population consisted of 3195 Duroc boars born from September 2011 to September 2016 on a single nucleus farm, with most of the pigs (3108 out of 3195) born from September 2011 to March 2014. The eight traits analyzed included age at 100 kg live weight (AGW, in days), average daily gain during 30–100 kg live weight (ADG, in g), back fat thickness at 100 kg live weight (BF, in mm), body judging score (BJS, ranging from 1 to 10), feed conversion ratio from 30 to 100 kg live weight (FCR), loin muscle area at 100 kg live weight (LMA, in mm^2^), loin muscle depth at 100 kg live weight (LMD, in mm), and total teat number (TN). Trait measurements began when the weight of pigs reached 30 ± 5 kg (average age 80 ± 8.4 days). Trait statistics are summarized in Additional file 1: Table S1.

The initial weight was measured 12 h after discontinuation of feeding. Single-space automatic feed intake recording equipment (FIRE, Osborne, KS, USA) was used to collect the feed intake and weight of the pigs. When a pig entered the measuring station, it was identified by radio frequency identification ear tags and the time and duration of each feeder visit, the weight of feed consumed per visit, and the cumulative feed consumed for each pig over a 24-h period were recorded. Daily feed intake was computed as the total feed intake during a 24-h period and daily weight each pig was computed as the average weight of the pig for the same 24-h period. Feeding was stopped in the afternoon of the day when the weight of each pig reached 100 ± 5 kg (average age 160 ± 9.0 days), and the final weight was recorded 12 h after feeding was stopped. Average daily gain was calculated based on daily gain from 30 to 100 kg live weight. The FCR from 30 to 100 kg was calculated by dividing average daily feed intake by ADG. A type B ultrasound scanner (SSD-500, Aloka, CT, USA) was used for measurements of muscle-related traits. Measurement positions were between the penultimate third and fourth ribs and five centimeters from the midline of the back on the left side, and the direction of the scanner head was perpendicular to the pig midline. The strong echogenic bands that appeared in the ultrasonic images and that represented the skin, connective tissue, and myolemma of the loin muscle were identified and provided a reliable basis for determining BF, LMA and LMD. TN was defined as the sum of the normal left and right teats counted within 48 h after birth. BJS was based on a ten-point scoring system and was recorded by skilled technicians for four body regions: head, foot and leg, forequarters, and hindquarters. A higher comprehensive BJS score is associated with a more desirable body shape and structure and is one of the company’s breeding aims in Duroc boars.

The phenotypic values of BJS had a skewed distribution and the Box-Cox transformation implemented in the R package [13] only changed the shape of the distribution slightly. However, since the original phenotypic values of BJS had a higher prediction accuracy than the transformed phenotypic values, we used the original phenotypic values. The original FCR values had outliers that resulted in a severely skewed phenotypic distribution, which became approximately normally distributed after removing outliers that were more than four standard deviations from the mean (see Additional file 1: Figure S1). Four traits (BF, LMA, LMD, and TN) had maximum values that were 4.02 to 4.25 standard deviations from the means, but these were not removed because the phenotypic distributions of these traits closely resembled a normal distribution (see Additional file 1: Figure S1).

### Whole-genome low-coverage sequencing and genotyping

Genomic DNA was extracted from ear tissues of 3195 Duroc boars using a DNeasy Blood & Tissue Kit (Qiagen 69506) and quantified using a NanoDrop spectrophotometer. Then, all DNA preparations were diluted to the same concentration in 96-well plates using a Qubit 2 Fluorometer (Invitrogen) and checked on a 1% agarose gel. The Tn5 transposase (Karolinska Institute 17177 Stockholm, Sweden) was used to construct the LCS libraries. The protocol and oligonucleotides for the Tn5 based library construction were as described previously [14, 15]. Two types of linker oligonucleotides were designed, separately, for the MGI and Illumina platforms. After PCR amplification using the KAPA HiFi HotStart ReadyMix (Roche), the products were quantified by Qubit 2 Fluorometric Quantitation and groups of 96 indexed samples were pooled in equal amounts. AMPure XP beads (Beckmann) was used to perform size-selection. The libraries were sequenced on a MGISEQ-2000 (PE 100) (192 libraries on 2 lanes) and a Illumina Hiseq Xten (PE 150) (84 libraries on one lane) sequencer. Each animal was sequenced at an average depth of 0.73 ± 0.17X and 96.7% of the reads were successfully mapped to the pig reference genome Sscrofa11.1. The BaseVar algorithm [16] was used to call SNP variants and estimate allele frequencies, and the STITCH algorithm [17] was used to impute SNPs.

A total of 11 million SNPs from the whole genome were obtained from imputation. Quality control of the SNP data consisted of removing SNPs with a minor allele frequency lower than 5% and those that did not pass the Hardy–Weinberg equilibrium test at p ≤ 10^−5^. After quality control, a clean SNP data set with 9,769,161 autosomal SNPs was subjected to further density reduction because haplotype reconstruction using nearly 10 million SNPs for many models (14 block sizes each with four haplotype models plus four haplotype models for gene-based haplotype blocks, i.e. 60 haplotype models) would be computationally too costly. Among the 9,769,161 autosomal SNPs, one SNP was selected from each 20-SNP window such that SNPs selected from adjacent windows were approximately equally spaced. Thus, 488,124 SNPs across the 18 pig autosomes were identified with a high average call rate of 98.9 ± 0.6% (see Additional file 1: Figure S2).

### Construction of haplotypes and haplotype blocks

For haplotype phasing, we used the Beagle 5.1 software [18] with default parameters and 30 phasing runs for each chromosome. Creation of haplotype blocks was based on fixed sizes in kilobases (kb), ranging from 50 to 5000 kb per block, and based on the location of genes. The method based on a fixed distance resulted in a greater number of haplotypes as block size increased, averaging from 16 haplotypes for 50 kb blocks to 696 haplotypes for 5000 kb blocks, while the average number of SNPs per block ranging from 14 for the 50-kb blocks to 1065 for the 5000-kb blocks (Table 1). Based on the Sscrofa genome annotation (ref_Sscrofa11.1_top level.gff3), 28,999 autosomal genes were available to construct the gene-based haplotype blocks, covering 1.28 Gb (56.5%) of the genome. Of these 28,999 genes, 26,319 had at least two SNPs, which were used to define gene-based haplotype blocks. To reduce variation in the size of the gene-based blocks, large genes were split into blocks of 200 to 500 kb, and the small genes (less than 50 kb), which accounted for 77.9% of the autosomal genes (see Additional file 1: Figure S3), were extended by 100 kb at each end. With these extensions, the gene-based haplotype blocks contained 364,643 SNPs (74.7% of the total number of SNPs). The size of the 26,319 gene-based haplotype blocks ranged from 0.6 to 1638.1 kb, with on average 13.9 SNPs per gene block, ranging from 2 to 602 (Table 2). After removing overlapping regions between haplotype blocks, the gene-based haplotype blocks covered 1.35 Gb (59.7%) of the autosomes.

**Table 1:** Statistics of haplotype blocks defined by fixed distance

	Size of haplotype block (kb)													
	50	100	150	200	250	300	350	500	750	1000	2000	3000	4000	5000
Total number of haplotypes	553,758	562,459	547,904	529,442	511,414	495,014	481,679	447,392	410,123	386,781	343,749	330,736	322,190	319,090
Number of blocks	33,629	18,726	13,060	10,044	8,177	6,890	5,961	4,252	2,881	2,185	1,110	752	565	458
Average number of haplotypes per block	16.47	30.04	41.95	52.71	62.54	71.85	80.81	105.22	142.35	177.02	309.68	439.81	570.25	696.70
Minimum SNPs per block	2	2	2	2	2	2	2	2	2	2	5	3	11	19
Maximum SNPs per block	79	120	167	195	227	285	320	414	549	774	1380	2081	2392	2840
Average number of SNPs per block	14.52	26.07	37.38	48.60	59.69	70.85	81.89	114.80	169.43	223.40	439.75	649.10	863.94	1065.78

**Table 2:** Statistics of haplotype blocks defined by gene boundaries

Total number of haplotypes	865,537
Number of blocks	26,319
Average number of haplotypes per block	32.89
Minimum SNPs per block	2
Maximum SNPs per block	602
Average number of SNPs per block	13.85
Minimum block distance (kb)	0.64
Maximum block distance (kb)	1638.08
Average distance per block (kb)	98.59

### Mixed model with SNP and haplotype effects for GBLUP and GREML

Each haplotype block was treated as a ‘locus’ and each haplotype within the haplotype block was treated as an ‘allele’ in the GVCHAP analysis [19]. The haplotypes in each block were converted into codes of haplotype genotypes for each boar using the GVCHAP pipeline [19]. Computation of genomic best linear unbiased prediction (GBLUP) of genetic values and genomic restricted maximum likelihood (GREML) estimation of variance components and heritabilities were conducted using the GVCHAP pipeline [19], which implements a multi-allelic mixed model. This model is based on a quantitative genetics model that results from the genetic partitioning of the genotypic values of the SNPs [20] and multi-allelic loci (haplotype blocks) [21] but implements genomic prediction and variance component estimation using a reparameterized and equivalent model due to the use of genomic relationship matrices of SNPs and/or haplotypes [19, 21, 22]. The mixed model based on the original quantitative genetics model for SNP and haplotype effects is:

1 $$\begin{aligned} {\mathbf{y}} & = {\mathbf{Xb}} + {\mathbf{Z}}\left( {{\mathbf{W}}_{\upalpha } {{\varvec{\upalpha}}}_{0} + {\mathbf{W}}_{{\updelta }} {{\varvec{\updelta}}}_{0} + {\mathbf{W}}_{{\upalpha {\text{h}}}} {{\varvec{\upalpha}}}_{{\text{oh}}} } \right) + {\mathbf{e}} \\ & = {\mathbf{Xb}} + {\mathbf{Z}}\left( {{\mathbf{a}} + {\mathbf{d}} + {\mathbf{a}}_{{\text{h}}} } \right) + {\mathbf{e}}, \\ \end{aligned}$$

where $\mathbf{Z}$ is an incidence matrix that allocates phenotypic observations to each individual, ${{\varvec{\upalpha}}}_{0}$ is a column vector of the additive effects of SNPs with incidence matrix ${\mathbf{W}}_{\upalpha }$, ${{\varvec{\updelta}}}_{0}$ is a column vector of the dominance effects of SNP genotypes with incidence matrix ${\mathbf{W}}_{\updelta }$, ${{\varvec{\upalpha}}}_{\text{oh}}$ is a column vector of the haplotype additive effects with incidence matrix ${\mathbf{W}}_{{\upalpha} {\text{h}}}$, $\mathbf{b}$ is a column vector of fixed year-season effects with incidence matrix $\mathbf{X}$, $\mathbf{a}={\mathbf{W}}_{{\upalpha }}{{\varvec{\upalpha}}}_{0}$ is a column vector of SNP additive values, $\mathbf{d}={\mathbf{W}}_{\updelta }{{\varvec{\updelta}}}_{0}$ is a column vector of SNP dominance values, ${\mathbf{a}}_{\text{h}}={\mathbf{W}}_{{\upalpha} {\text{h}}}{{\varvec{\upalpha}}}_{0\text{h}}$ is a column vector of haplotype additive values, and $\mathbf{e}$ is a column vector of random residuals. The SNP coding in ${\mathbf{W}}_{{\upalpha }}$ and ${\mathbf{W}}_{\updelta }$ is the same as the quantitative genetic coding for SNPs [20], and the haplotype coding in ${\mathbf{W}}_{{\upalpha} {\text{h}}}$ is the same as the multi-allelic coding based on genetic partitions [21]. The reparameterized and equivalent model of Eq. (1) due to the use of genomic relationships is:

2 $$\begin{aligned} {\mathbf{y}} & = {\mathbf{Xb}} + {\mathbf{Z}}\left( {{\mathbf{T}}_{{\upalpha }} {{\varvec{\upalpha}}} + {\mathbf{T}}_{\updelta } {{\varvec{\updelta}}} + {\mathbf{T}}_{{{\upalpha} {\text{h}}}} {{\varvec{\upalpha}}}_{{\text{h}}} } \right) + {\mathbf{e}} \hfill \\ & = {\mathbf{Xb}} + {\mathbf{Z}}\left( {{\mathbf{a}} + {\mathbf{d}} + {\mathbf{a}}_{{\text{h}}} } \right) + {\mathbf{e}}, \hfill \\ \end{aligned}$$

where ${\mathbf{T}}_{{\upalpha }}={\mathbf{W}}_{{\upalpha }}/{\text{k}}_{{\upalpha }}^{1/2}$, ${\mathbf{T}}_{\updelta }={\mathbf{W}}_{\updelta }/{\text{k}}_{\updelta }^{1/2}$, ${\mathbf{T}}_{{\upalpha} {\text{h}}}={\mathbf{W}}_{{\upalpha} {\text{h}}}/{\text{k}}_{{\upalpha} {\text{h}}}^{1/2}$; and ${\text{k}}_{{\upalpha }}=\text{tr}({\mathbf{W}}_{{\upalpha }}{\mathbf{W}}_{{\upalpha }}^{{^{\prime}}})/\text{n}$, ${\text{k}}_{\updelta }=\text{tr}({\mathbf{W}}_{\updelta }{\mathbf{W}}_{\updelta }^{{^{\prime}}})/\text{n}$, ${\text{k}}_{{\upalpha} {\text{h}}}=\text{tr}({\mathbf{W}}_{{\upalpha} {\text{h}}}{\mathbf{W}}_{{\upalpha} {\text{h}}}^{{^{\prime}}})/\text{n}$, and where $\text{n}$ is the number of individuals. The first moment is $\text{E}\left(\mathbf{y}\right)=\mathbf{X}\mathbf{b}$, and the second moments resulting from the reparameterized and equivalent model are:

3 $$\text{var}\left(\mathbf{a}\right)={\upsigma }_{{\upalpha }}^{2}{\mathbf{T}}_{{\upalpha }}{\mathbf{T}}_{{\upalpha }}^{{^{\prime}}}={\upsigma }_{{\upalpha }}^{2}{\mathbf{A}}_{\text{g}}={\upsigma }_{{\upalpha }}^{2}{\mathbf{W}}_{{\upalpha }}{\mathbf{W}}_{{\upalpha }}^{{^{\prime}}}/{\text{k}}_{{\upalpha }}={\mathbf{G}}_{\text{a}},$$

4 $$\text{var}\left(\mathbf{d}\right)={\upsigma }_{\updelta }^{2}{\mathbf{T}}_{\updelta }{\mathbf{T}}_{\updelta }^{{^{\prime}}}={\upsigma }_{\updelta }^{2}{\mathbf{D}}_{\text{g}}={\upsigma }_{\updelta }^{2}{\mathbf{W}}_{\updelta }{\mathbf{W}}_{\updelta }^{{^{\prime}}}/{\text{k}}_{\updelta }={\mathbf{G}}_{\text{d}},$$

5 $$\text{var}\left({\mathbf{a}}_{\text{h}}\right)={\mathbf{T}}_{{\upalpha} {\text{h}}}{\mathbf{T}}_{{\upalpha} {\text{h}}}^{{^{\prime}}}={\upsigma }_{{\upalpha} {\text{h}}}^{2}{\mathbf{A}}_{\text{gh}}={\upsigma }_{{\upalpha} {\text{h}}}^{2}{\mathbf{W}}_{{\upalpha} {\text{h}}}{\mathbf{W}}_{{\upalpha} {\text{h}}}^{{^{\prime}}}/{\text{k}}_{{\upalpha} {\text{h}}}={\mathbf{G}}_{\text{ah}},$$

6 $$\text{var}\left(\mathbf{y}\right)=\mathbf{Z}\left({\upsigma }_{{\upalpha }}^{2}{\mathbf{A}}_{\text{g}}+{\upsigma }_{\updelta }^{2}{\mathbf{D}}_{\text{g}}+{\upsigma }_{{\upalpha} {\text{h}}}^{2}{\mathbf{A}}_{\text{gh}}\right){\mathbf{Z}}^{^{\prime}}+{\upsigma }_{\text{e}}^{2}{\mathbf{I}}_{\text{N}}=\mathbf{V}$$

where ${\upsigma }_{{\upalpha }}^{2}$, ${\upsigma }_{\updelta }^{2}$, and ${\upsigma }_{{\upalpha} {\text{h}}}^{2}$ are the SNP additive variance, the SNP dominance variance, and the haplotype additive variance, respectively; ${\mathbf{A}}_{\text{g}}$ is the SNP additive relationship matrix; ${\mathbf{D}}_{\text{g}}$ is the SNP dominance relationship matrix; ${\mathbf{A}}_{\text{gh}}$ is the haplotype additive relationship matrix; ${\upsigma }_{\text{e}}^{2}$ is the residual variance; and $\mathbf{V}$ is the phenotypic variance–covariance matrix. The GVCHAP program first estimates the variance components of ${\upsigma }_{{\upalpha }}^{2}$, ${\upsigma }_{\updelta }^{2}$, and ${\upsigma }_{{\upalpha} {\text{h}}}^{2}$ in Eqs. (3) to (6) and the corresponding heritabilities using GREML, and then computes GBLUP and associated reliability estimates [19, 21].

### Evaluation of the prediction accuracy of haplotype models using cross-validation

Ten-fold cross-validation was used to evaluate the accuracy of predicting phenotypic values. The 3195 Duroc pigs were randomly divided into ten validation data sets of 320 pigs each, except for the 10th set, which had 315 pigs. Phenotypic observations of individuals in the validation set were omitted in the calculation of the GBLUP. The following six predictions were evaluated for each validation set for each method of haplotype blocking and for each trait:

- Model-1: SNP additive and dominance, and haplotype additive values ($\text{A}+\text{D}+\text{H}$);
- Model-2: SNP and haplotype additive values ($\text{A}+\text{H}$);
- Model-3: SNP dominance values and haplotype additive values ($\text{D}+\text{H}$);
- Model-4: haplotype additive values ($\text{H}$);
- Model-5: SNP additive and dominance values ($\text{A}+\text{D}$);
- Model-6: SNP additive values ($\text{A}$).

Models-1 to -4 contain haplotype additive values, while Model-5 and Model-6 contain only SNP predictions. The comparison of prediction accuracies of Model-1 to Model-4 with Model-5 and Model-6, therefore, evaluates whether the use of haplotypes improves prediction accuracy.

Prediction accuracy was estimated as the correlation between the phenotypic values and the predicted genetic values in each validation population [8, 11, 23–28] and averaged over the 10 validation populations. Thus, prediction accuracy here refers to the observed accuracy of predicting phenotypic values. Observed prediction accuracies were computed for both the original phenotypic values and phenotypic values corrected for fixed year-season effects estimated from each of the 10 training data sets. The fixed effects for each training population were estimated using the best linear unbiased estimation (BLUE) method which is also a generalized least squares (GLS) estimation [22, 29] implemented in GVCHAP [19]. Note that, phenotypic values in the training population are automatically corrected for fixed effects when calculating GBLUP [20, 21]. The observed accuracy of predicting phenotypic values was calculated as:

7 $${\hat{\text{R}}}_{{0}_{\text{jp}}}=\text{corr}\left({\hat{\text{g}}}_{{0}_{\text{j}}},{\text{y}}_{0}\right)=\left[{\sum }_{\text{k}=1}^{10}\text{corr}\left({\hat{\text{g}}}_{{0}_{\text{jk}}},{\text{y}}_{{0}_{\text{k}}}\right)\right]/10,$$

where ${\hat{\text{R}}}_{{0}_{\text{jp}}}$ is the observed accuracy for predicting the phenotypic values (or predictive ability [23]), ${\hat{\text{g}}}_{{0}_{\text{j}}}$ is the GBLUP of ${\text{g}}_{{0}_{\text{j}}}$, ${\text{g}}_{{0}_{\text{j}}}$ is the unobservable genetic values, ${\text{y}}_{0}$ are the phenotypic observations, subscript ‘$0$’ denotes validation population, ‘$\text{corr}$’ stands for correlation, and $\text{j}$ represents the total genetic values under Model-$\text{j}$, $\text{j}=1,\dots ,6$. In addition to the observed accuracy, two theoretical measures of accuracy that do not involve the phenotypic observations were also calculated: the theoretical accuracy for predicting phenotypic values [23, 24], and the theoretical accuracy of predicted genetic values as the square root of the reliability under the SNP and haplotype models [19].

The theoretical accuracy of predicting the genetic value of the $\text{i}$-th training or validation individual for $\hat{\mathbf{g}}=\hat{\mathbf{a}}+\hat{\mathbf{d}}+{\hat{\mathbf{a}}}_{\text{h}}$ of Model-1 was calculated as the square root of the reliability implemented by GVCHAP [19]:

8 $${\text{R}}_{\text{gi}}={\left\{\frac{{\left(\begin{aligned}{\mathbf{G}}_{{\upalpha}}{\mathbf{Z}^{\prime}}\mathbf{PZ}{\mathbf{G}}_{{\upalpha }}+{\mathbf{G}}_{\updelta}{\mathbf{Z}^{\prime}}\mathbf{PZ}{\mathbf{G}}_{\updelta }+{\mathbf{G}}_{{\upalpha}{\text{h}}}{\mathbf{Z}^{\prime}}\mathbf{PZ}{\mathbf{G}}_{{\upalpha} {\text{h}}}\\{+\mathbf{G}}_{{\upalpha }}{\mathbf{Z}^{\prime}}\mathbf{PZ}{\mathbf{G}}_{\updelta}+{\mathbf{G}}_{\updelta }{\mathbf{Z}^{\prime}}\mathbf{PZ}{\mathbf{G}}_{{\upalpha}}+{\mathbf{G}}_{{\upalpha }}{\mathbf{Z}^{\prime}}\mathbf{PZ}{\mathbf{G}}_{{\upalpha}{\text{h}}}\\ {+\mathbf{G}}_{{\upalpha}{\text{h}}}{\mathbf{Z}^{\prime}}\mathbf{PZ}{\mathbf{G}}_{{\upalpha}}+{\mathbf{G}}_{\updelta }{\mathbf{Z}^{\prime}}\mathbf{PZ}{\mathbf{G}}_{{\upalpha}{\text{h}}}+{\mathbf{G}}_{{\upalpha}{\text{h}}}{\mathbf{Z}^{\prime}}\mathbf{PZ}{\mathbf{G}}_{\updelta}\end{aligned}\right)}_{\text{ii}}}{\left({\text{A}}_{\text{g}}^{\text{ii}}{\upsigma }_{{\upalpha}}^{2}+{\text{D}}_{\text{g}}^{\text{ii}}{\upsigma }_{\updelta}^{2}+{\text{A}}_{\text{gh}}^{\text{ii}}{\upsigma }_{{\upalpha} {\text{h}}}^{2}\right)}\right\}}^{1/2},$$

where $\mathbf{P}={\mathbf{V}}^{-1}-{\mathbf{V}}^{-1}\mathbf{X}{({\mathbf{X}}^{\mathbf{^{\prime}}}{\mathbf{V}}^{-1}\mathbf{X})}^{-}{\mathbf{X}}^{\mathbf{^{\prime}}}{\mathbf{V}}^{-1}$, and ${\text{A}}_{\text{g}}^{\text{ii}}$, ${\text{D}}_{\text{g}}^{\text{ii}}$ and ${\text{A}}_{\text{gh}}^{\text{ii}}$ are the $\text{i}$-th diagonal elements of ${\mathbf{A}}_{\text{g}}$, ${\mathbf{A}}_{\text{g}}$ and ${\mathbf{A}}_{\text{gh}}$ [Eqs. (3) to (6)], respectively. The accuracy for Model-2 to Model-6 can be readily derived from Eq. (8), e.g., the accuracy for $\hat{\mathbf{g}}=\hat{\mathbf{a}}+{\hat{\mathbf{a}}}_{\text{h}}$ of Model-2 is obtained from Eq. (8) by deleting all terms involving ‘$\updelta$’. In the following, a subscript ‘$0$’ is added to Eq. (8) to denote the validation population, and ‘$\text{g}$’ is changed to ‘$\text{j}$’ to indicate Model-$\text{j}$, $\text{j}=1,\dots ,6$. The theoretical accuracy for predicting the genetic values in the tenfold validation study was calculated as the average of the ${\text{R}}_{{0}_{\text{ji}}}$ values of all individuals in each validation population and then averaged over all 10 validation populations., i.e.:

9 $${\text{R}}_{{0}_{\text{j}}}=\text{corr}\left({\hat{\text{g}}}_{{0}_{\text{j}}},{\text{g}}_{{0}_{\text{j}}}\right)=\left[\sum_{\text{k}=1}^{10}\left(\sum_{\text{i}=1}^{{\text{n}}_{{0}_{\text{k}}}}{\text{R}}_{{0}_{\text{ji}}}^{\text{k}}\right)/{\text{n}}_{{0}_{\text{k}}}\right]/10,$$

where ${\text{R}}_{{0}_{\text{ji}}}^{\text{k}}$ is the value of ${\text{R}}_{{0}_{\text{ji}}}$ and ${\text{n}}_{{0}_{\text{k}}}$ is the number of individuals in the $\text{k}$-th validation population.

The theoretical accuracy for predicting phenotypic values was calculated as:

10 $${\text{R}}_{{0}_{\text{jp}}}={\text{R}}_{{0}_{\text{j}}}\sqrt{{\text{h}}_{\text{g}}^{2}}=\left[\sum_{\text{k}=1}^{10}{\text{R}}_{{0}_{\text{jk}}}\sqrt{{\text{h}}_{\text{gk}}^{2}}\right]/10,$$

where ${\text{R}}_{{0}_{\text{jp}}}$ is the theoretical accuracy for predicting phenotypic values, ${\text{h}}_{\text{g}}^{2}$ is the total genomic heritability for Model-j, ${\text{R}}_{{0}_{\text{j}}}$ is the theoretical accuracy for predicting genetic values of individuals in the validation population, calculated based on the square root of the reliability from the GVCHAP output file using the estimated ${\text{h}}_{\text{g}}^{2}$ from each validation population (${\hat{\text{h}}}{_{\text{gk}}^{2}}$) for ${\text{h}}_{\text{gk}}^{2}$ in Eq. (10).

Depending on the prediction model, ${\text{h}}_{\text{g}}^{2}$ has one of the following expressions:

11 $${\hat{\text{h}}}{_{{\text{g}}}^{2}} = {\hat{\text{h}}}{_{{{\upalpha} {\text{s}}}}^{2}} + {\hat{\text{h}}}{_{{\updelta {\text{s}}}}^{2}} + {\hat{\text{h}}}{_{{{\upalpha} {\text{h}}}}^{2}}\; {\text{for Model-}}1,$$

12 $${\hat{\text{h}}}{_{{\text{g}}}^{2}} = {\hat{\text{h}}}{_{{{\upalpha} {\text{s}}}}^{2}} + {\hat{\text{h}}}{_{{{\upalpha} {\text{h}}}}^{2}} \;{\text{for Model-}}2,$$

13 $${\hat{\text{h}}}{_{{\text{g}}}^{2}} = {\hat{\text{h}}}{_{{\updelta {\text{s}}}}^{2}} + {\hat{\text{h}}}{_{{{\upalpha} {\text{h}}}}^{2}} \;{\text{for Model-}}3,$$

14 $${\hat{\text{h}}}{_{{\text{g}}}^{2}} = {\hat{\text{h}}}{_{{{\upalpha} {\text{h}}}}^{2}} \;{\text{for Model-}}4,$$

15 $${\hat{\text{h}}}{_{{\text{g}}}^{2}} = {\hat{\text{h}}}{_{{\text{s}}}^{2}} = {\hat{\text{h}}}{_{{{\upalpha }2}}^{2}} + {\hat{\text{h}}}{_{\updelta }^{2}} \;{\text{for Model-}}5,$$

16 $${\hat{\text{h}}}{_{{\text{g}}}^{2}} = {\hat{\text{h}}}{_{{\text{s}}}^{2}} = {\hat{\text{h}}}{_{{{\upalpha }1}}^{2}} \; {\text{for Model-}}6$$

where ${\hat{\text{h}}}{_{{\upalpha }1}^{2}}$ is the estimate of the SNP additive heritability from Model-6, ${\hat{\text{h}}}{_{{\upalpha }2}^{2}}$ is the estimate of the SNP additive heritability from Model-5, ${\hat{\text{h}}}{_{\updelta }^{2}}$ is the estimate of the SNP dominance heritability from Model-5, ${\hat{\text{h}}}{_{{\upalpha} {\text{s}}}^{2}}$ is the estimate of the SNP additive heritability from Model-1 or Model-2, ${\hat{\text{h}}}{_{{\updelta} {\text{s}}}^{2}}$ is the estimate of the SNP dominance heritability from Model-1 or Model-3, ${\hat{\text{h}}}{_{{\upalpha} {\text{h}}}^{2}}$ is the estimate of the haplotype additive heritability from Model-1 to Model-4, and ${\hat{\text{h}}}{_{\text{s}}^{2}}$ is the estimate of the total SNP heritability of Model-5 or Model-6.

### Estimation of the haplotype epistasis heritability

The estimate of the haplotype epistasis heritability ${(\hat{\text{h}}}{_{\text{E}}^{2}}$) was defined as the difference between the estimates of total heritability of the haplotype models (Model-1 to Model-4) ${(\hat{\text{h}}}{_{\text{g}}^{2}})$ and the total heritability of the corresponding SNP models (Model-5 and Model-6) (${\hat{\text{h}}}{_{\text{s}}^{2}})$, i.e., ${\hat{\text{h}}}{_{\text{E}}^{2}}={\hat{\text{h}}}{_{\text{g}}^{2}}-{\hat{\text{h}}}{_{\text{s}}^{2}}.$ This difference measures the genetic variance generated by haplotypes that are unavailable from the SNP additive or dominance variance and was shown to be responsible for the increased prediction accuracy of haplotype models [11]. Depending on the SNP and haplotype prediction models, four sets of ${\hat{\text{h}}}{_{\text{E}}^{2}}$ expressions were defined, as described previously [11], i.e.:

17 $${\hat{\text{h}}}{_{{\text{E}}}^{2}} = {\hat{\text{h}}}{_{{\text{g}}}^{2}} - {\hat{\text{h}}}{_{{\text{s}}}^{2}} = {\hat{\text{h}}}{_{{{\upalpha} {\text{h}}}}^{2}} - {\hat{\text{h}}}{_{{{\upalpha }1}}^{2}} \;{\text{for Model-4}},$$

18 $${\hat{\text{h}}}{_{{\text{E}}}^{2}} = {\hat{\text{h}}}{_{{\text{g}}}^{2}} - {\hat{\text{h}}}{_{{\text{s}}}^{2}} = \left( {{\hat{\text{h}}}{_{{{\upalpha} {\text{h}}}}^{2}} + {\hat{\text{h}}}{_{{{\upalpha} {\text{s}}}}^{2}} } \right) - {\hat{\text{h}}}{_{{{\upalpha }1}}^{2}} \;{\text{for Model-2}},$$

19 $${\hat{\text{h}}}{_{{\text{E}}}^{2}} = {\hat{\text{h}}}{_{{\text{g}}}^{2}} - {\hat{\text{h}}}{_{{\text{s}}}^{2}} = \left( {{\hat{\text{h}}}{_{{{\upalpha} {\text{h}}}}^{2}} + {\hat{\text{h}}}{_{{{\upalpha} {\text{s}}}}^{2}} + {\hat{\text{h}}}{_{{\updelta {\text{s}}}}^{2}} } \right) - ({\hat{\text{h}}}{_{{{\upalpha }2}}^{2}} + {\hat{\text{h}}}{_{\updelta }^{2}} )\;{\text{for Model-1}},$$

20 $${\hat{\text{h}}}{_{{\text{E}}}^{2}} = {\hat{\text{h}}}{_{{\text{g}}}^{2}} - {\hat{\text{h}}}{_{{\text{s}}}^{2}} = \left( {{\hat{\text{h}}}{_{{{\upalpha} {\text{h}}}}^{2}} + {\hat{\text{h}}}{_{{\updelta {\text{s}}}}^{2}} } \right) - ({\hat{\text{h}}}{_{{{\upalpha }2}}^{2}} + {\hat{\text{h}}}{_{\updelta }^{2}} )\;{\text{for Model-3}},$$

where ${\hat{\text{h}}}{_{{\upalpha} {\text{h}}}^{2}}$, ${\hat{\text{h}}}{_{{\upalpha }1}^{2}}$, ${\hat{\text{h}}}{_{{\upalpha }2}^{2}}$, ${\hat{\text{h}}}{_{\updelta }^{2}}$, ${\hat{\text{h}}}{_{{\upalpha} {\text{s}}}^{2}}$ and ${\hat{\text{h}}}{_{{\updelta} {\text{s}}}^{2}}$ have the same definitions as in Eqs. (11) to (16). The heritability estimates on the right-hand sides of Eqs. (17) to (20) are available from the GREML output files of GVCHAP [19]. Relative haplotype epistasis heritability was defined as the ratio of the haplotype epistasis heritability to the SNP additive heritability to serve as a measure of the size of the haplotype epistasis heritability relative to the SNP additive heritability. Depending on the haplotype prediction model, estimates of relative haplotype epistasis heritability were obtained as:

21 $${\hat{\text{h}}}{_{{{\text{Er}}}}^{2}} = {\hat{\text{h}}}{_{{\text{E}}}^{2}} /{\hat{\text{h}}}{_{{\upalpha 1}}^{2}} \;{\text{for Model-2 and Model-4}},$$

22 $${\hat{\text{h}}}{_{{{\text{Er}}}}^{2}} = {\hat{\text{h}}}{_{{\text{E}}}^{2}} /{\hat{\text{h}}}{_{{\upalpha 2}}^{2}} \;{\text{for Model-1 and Model-3}}.$$

To assess the impact of relative haplotype epistasis heritability on the increase in prediction accuracy, the Pearson’s correlation coefficient between estimates of relative haplotype epistasis heritability [Eqs. (21) and (22)] and the increase in prediction accuracy due to haplotypes was calculated and tested for statistical significance. For comparison, correlation coefficients between the prediction accuracy and estimates of SNP additive heritability, SNP total heritability, and the total heritability based on SNPs and haplotypes were also calculated and tested for each trait.

### Profiles of heritability estimates for SNPs and haplotype blocks

Here, a heritability profile is a Manhattan plot of heritability estimates for SNPs or haplotype blocks using the SNPEVG2 program [30], where the heritability estimate for each SNP or each haplotype block was from the GREML output file from GVCHAP [19]. The heritability estimate for each SNP is the contribution of the SNP to the phenotypic variance and is also the contribution to the SNP additive or dominance heritability [31], and the heritability estimate for each haplotype block is the contribution of the haplotype block to the phenotypic variance and is also the contribution to the haplotype additive heritability [21], i.e.:

23 $${\hat{\text{h}}}{_{{\upalpha} {\text{i}}}^{2}}={\hat{\upsigma }}{_{{\upalpha} {\text{i}}}^{2}}/{\hat{\upsigma }}{_{\text{y}}^{2}}=\left({\hat{{\upalpha }}}{_{\text{i}}^{2}}/\sum_{\text{i}=1}^{\text{m}}{\hat{{\upalpha }}}{_{\text{i}}^{2}}\right){\hat{\text{h}}}{_{{\upalpha }}^{2}}=\left({\hat{{\upalpha }}}{_{\text{i}}^{2}}/{\hat{{\varvec{\upalpha}}}}^{\prime}\hat{{\varvec{\upalpha}}}\right){\hat{\text{h}}}{_{{\upalpha }}^{2}},$$

24 $${\hat{\text{h}}}{_{{\updelta} {\text{i}}}^{2}}={\hat{\upsigma }}{_{{\updelta} {\text{i}}}^{2}}/{\hat{\upsigma }}{_{\text{y}}^{2}}=\left({\hat{\updelta }}{_{\text{i}}^{2}}/\sum_{\text{i}=1}^{\text{m}}{\hat{\updelta }}{_{\text{i}}^{2}}\right){\hat{\text{h}}}{_{\updelta }^{2}}=\left({\hat{\updelta }}{_{\text{i}}^{2}}/{\hat{{\varvec{\updelta}}}}^{\prime}\hat{{\varvec{\updelta}}}\right){\hat{\text{h}}}{_{\updelta }^{2}},$$

25 $${\hat{\text{h}}}{_{{\upalpha} {\text{hi}}}^{2}}={\hat{\upsigma }}{_{{\upalpha} {\text{hi}}}^{2}}/{\hat{\upsigma }}{_{\text{y}}^{2}}=\left({\hat{{\upalpha }}}{_{\text{hi}}^{2}}/\sum_{\text{i}=1}^{\text{b}}{\hat{{\upalpha }}}{_{\text{hi}}^{2}}\right){\hat{\text{h}}}{_{{\upalpha} {\text{h}}}^{2}}=\left({\hat{{\upalpha }}}{_{\text{hi}}^{2}}/{\hat{{\varvec{\upalpha}}}}{_{\text{h}}^{\prime}}{\hat{{\varvec{\upalpha}}}}_{\text{h}}\right){\hat{\text{h}}}{_{{\upalpha} {\text{h}}}^{2}},$$

where ${\hat{\text{h}}}{_{{\upalpha} {\text{i}}}^{2}}$, ${\hat{\upsigma }}{_{{\upalpha} {\text{i}}}^{2}}$ and ${\hat{{\upalpha }}}_{\text{i}}$ are the additive heritability, variance and effect of the $\text{i}$-th SNP;${\hat{\text{h}}}{_{{\updelta} {\text{i}}}^{2}}$, ${\hat{\upsigma }}{_{{\updelta} {\text{i}}}^{2}}$ and ${\hat{\updelta }}_{\text{i}}$ are the dominance heritability, variance and effect of the $\text{i}$-th SNP;${\hat{\text{h}}}{_{{\upalpha} {\text{hi}}}^{2}}$, ${\hat{\upsigma }}{_{{\upalpha} {\text{hi}}}^{2}}$ and ${\hat{{\upalpha }}}_{\text{hi}}$ are the haplotype additive heritability, variance and effect of the $\text{i}$-th haplotype block with $\text{b}$ being the number of haplotype blocks, respectively; ${\hat{\upsigma }}{_{\text{y}}^{2}}$ is the phenotypic variance and is equal to ${\hat{\upsigma }}{_{{\upalpha }}^{2}}+{\hat{\upsigma }}{_{\updelta }^{2}}+{\hat{\upsigma }}{_{{\upalpha} {\text{h}}}^{2}}+{\hat{\upsigma }}{_{\text{e}}^{2}}$, ${\hat{\text{h}}}{_{{\upalpha }}^{2}}={\hat{\upsigma }}{_{{\upalpha }}^{2}}/{\hat{\upsigma }}{_{\text{y}}^{2}}$ is the genomic SNP additive heritability, ${\hat{\text{h}}}{_{\updelta }^{2}}={\hat{\upsigma }}{_{\updelta }^{2}}/{\hat{\upsigma }}{_{\text{y}}^{2}}$ is the genomic SNP dominance heritability, ${\hat{\text{h}}}{_{{\upalpha} {\text{h}}}^{2}}={\hat{\upsigma }}{_{{\upalpha} {\text{h}}}^{2}}/{\hat{\upsigma }}{_{\text{y}}^{2}}$ is the genomic haplotype additive heritability. It can be readily seen that the sum of all SNP or haplotype heritability estimates is the genomic SNP or haplotype heritability, i.e., $\sum_{\text{i}=1}^{\text{m}}{\hat{\text{h}}}{_{{\upalpha} {\text{i}}}^{2}}={\hat{\text{h}}}{_{{\upalpha }}^{2}}$, $\sum_{\text{i}=1}^{\text{m}}{\hat{\text{h}}}{_{{\updelta} {\text{i}}}^{2}}={\hat{\text{h}}}{_{\updelta }^{2}}$, $\sum_{\text{i}=1}^{\text{b}}{\hat{\text{h}}}{_{{\upalpha} {\text{hi}}}^{2}}={\hat{\text{h}}}{_{{\upalpha} {\text{h}}}^{2}}$. Equations (23) to (25) can be shown using the example of SNP additive heritability. The additive variances of $\text{m}$ SNPs and the $\text{i}$-th SNP can be estimated as:

26 $$\begin{aligned} {\hat{\upsigma }}{_{{\upalpha }}^{2}} & = {{\hat{\mathbf{\upalpha }}^{\prime}}}{\hat{\mathbf{\upalpha }}}/[{\text{m}} - {\text{tr}}({\mathbf{C}}^{{\upalpha \upalpha }} ){\uplambda }_{\upalpha } ] \hfill \\ & = \mathop \sum \limits_{{{\text{i}} = 1}}^{{\text{m}}} \hat{\upalpha} _{{\text{i}}}^{2} /[{\text{m}} - {\text{tr}}({\mathbf{C}}^{{\upalpha \upalpha }} ){\uplambda }_{\upalpha } ] = \mathop \sum \limits_{{{\text{i}} = 1}}^{{\text{m}}} {\hat{\upsigma }}{_{{\upalpha {\text{i}}}}^{2}} , \hfill \\ \end{aligned}$$

27 $${\hat{\upsigma }}{_{{\upalpha} {\text{i}}}^{2}}={\hat{{\upalpha }}}{_{\text{i}}^{2}}/\text{m}-\text{tr}({\mathbf{C}}^{{\upalpha \upalpha }}){\uplambda }_{{\upalpha }},$$

where ${\mathbf{C}}^{{\upalpha \upalpha }}$ is the submatrix in the inverse or generalized inverse of the coefficient matrix of the mixed model equations (MME) corresponding to the SNP additive effects, and ${\uplambda }_{{\upalpha }}={\hat{\upsigma }}{_{\text{e}}^{2}}/{\hat{\upsigma }}{_{{\upalpha }}^{2}}$. Dividing Eq. (27) by ${\hat{\upsigma }}{_{\text{y}}^{2}}$ and multiplying by ${\hat{\upsigma }}{_{{\upalpha }}^{2}}/{\hat{\upsigma }}{_{{\upalpha }}^{2}}$ yields Eq. (23), i.e.:

$$\begin{aligned} {\hat{\text{h}}}{_{{{\upalpha} {\text{i}}}}^{2}} & = \left( {{\hat{\upsigma }}{_{{{\upalpha} {\text{i}}}}^{2}} /{\hat{\upsigma }}{_{{\text{y}}}^{2}} } \right)\left( {{\hat{\upsigma }}{_{{\upalpha }}^{2}} /{\hat{\upsigma }}{_{{\upalpha }}^{2}} } \right) = \left( {{\hat{\upsigma }}{_{{{\upalpha} {\text{i}}}}^{2}} /{\hat{\upsigma }}{_{{\upalpha }}^{2}} } \right)\left( {{\hat{\upsigma }}{_{{\upalpha }}^{2}} /{\hat{\upsigma }}{_{{\text{y}}}^{2}} } \right) \hfill \\ & = \left( {{\hat{\upalpha }}{_{{\text{i}}}^{2}} /\mathop \sum \limits_{{{\text{i}} = 1}}^{{\text{m}}} {\hat{\upalpha }}{_{{\text{i}}}^{2}} } \right){\hat{\text{h}}}{_{{\upalpha }}^{2}} = \left( {{\hat{\upalpha }}{_{{\text{i}}}^{2}} /{\hat{\varvec{\upalpha }}}^{^{\prime}} {\hat{\varvec{\upalpha }}}} \right){\hat{\text{h}}}{_{{\upalpha }}^{2}} . \hfill \\ \end{aligned}$$

Equations (24) and (25) can be shown similarly. Note that Eqs. (26) and (27) using MME are not implemented by GVCHAP but are convenient for proving Eq. (23) and yield identical results as the conditional expectation (CE) method implemented by GVCHAP. The CE method is more efficient than the MME method when the number of genetic effects is greater than the number of individuals [20, 21]. With genome-wide haplotypes in the prediction model, the number of genetic effects should generally be much larger than the number of individuals. In this study, the number of SNPs was 488,124, the number of haplotypes ranged from 319,090 to 553,758 for haplotype blocks using fixed chromosome distances (Table 1) and was 865,537 for gene-based haplotype blocks (Table 2), whereas the number of individuals was 3195. For this type of data structure, the MME method for estimating genetic effects and their variances is computationally prohibitive, and the CE method is computationally feasible.

The heritability size for a SNP is related to the number of SNPs in the model, i.e., the larger the number of SNPs, the smaller the heritability estimate for each SNP [24, 32]. Consequently, the heritability for a SNP is not comparable with the heritability for a haplotype block. However, the SNP heritability estimates from Eqs. (23) and (24) are comparable regarding their sizes, and the haplotype additive heritability estimates from Eq. (25) are also comparable regarding their sizes. Therefore, the heritability profile for SNPs or haplotypes provides a global view of the relative genetic contributions of the different genes and chromosome locations to the phenotype. The difference between heritability profiles for SNPs and haplotypes was used to assess the likely reason for the success or failure of haplotype models.

## Results and discussion

### Impact of using haplotypes on prediction accuracy

**Fig. 1:**
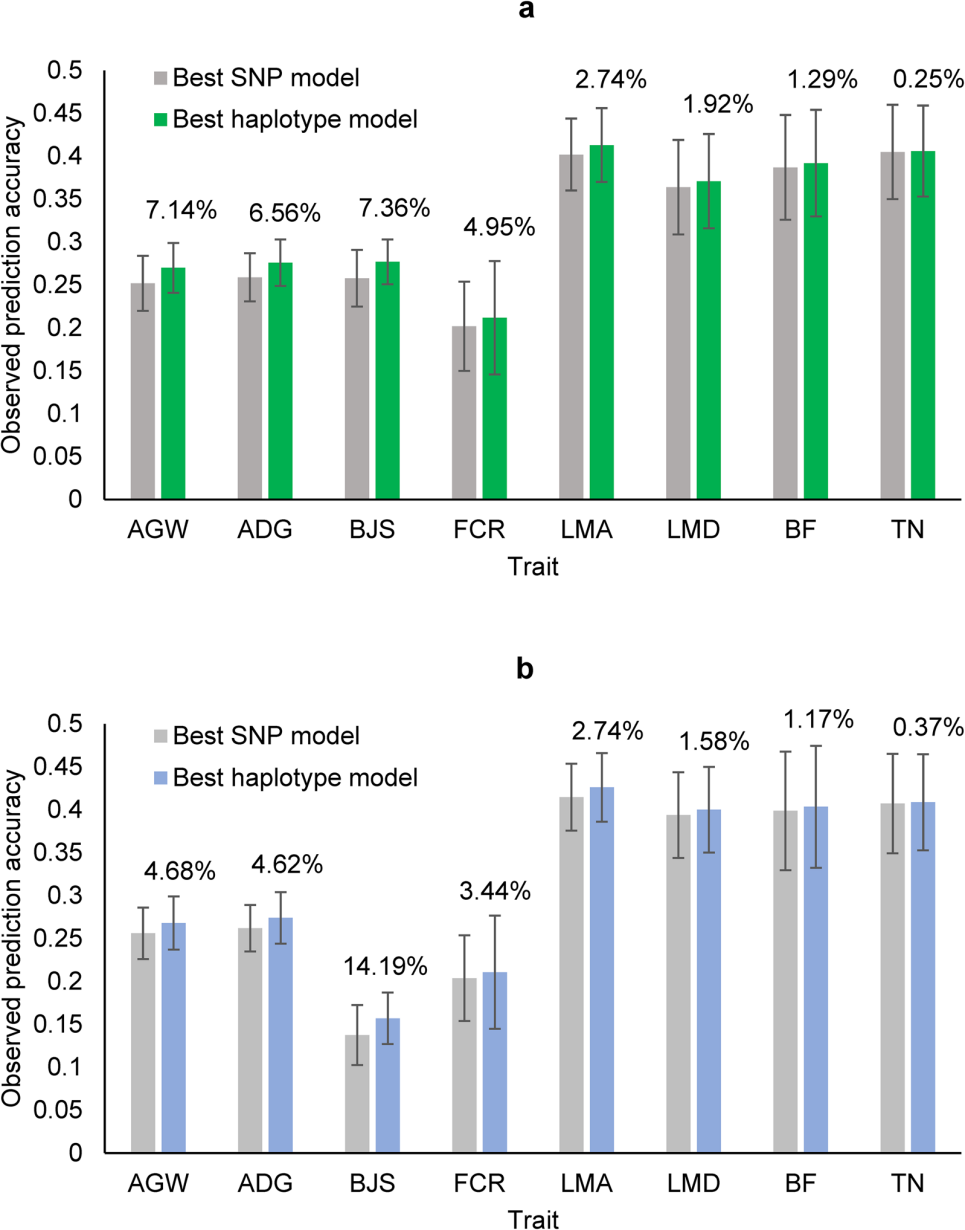
Observed prediction accuracy of the best haplotype model relative to the best SNP model for predicting phenotypic values of each trait from ten-fold validations. **a** Observed prediction accuracy using the original phenotypic values of the validation populations. **b** Observed prediction accuracy using the corrected phenotypic values of the validation populations. The error bar is one standard deviation above and below the average prediction accuracy, where standard deviation was calculated from tenfold validations. *AGW* age at 100 kg live weight, *ADG* daily gain during, *BJS* body judging score, *FCR* Feed conversion ratio, *LMA* loin muscle area at 100 kg, *LMD* loin muscle depth at 100 kg, *BF* back fat thickness at 100 kg, *TN* teat number

We found that, for the eight traits included in this study, prediction accuracy was improved by using haplotypes in the prediction model, for both prediction of the original (Fig. 1a) and the corrected phenotypic values with removal of the fixed year-season effects (Fig. 1b), except for TN, for which the increase in accuracy was negligible. The increase in prediction accuracy due to the use of haplotypes relative to the prediction accuracy of the best SNP model (additive only, or additive and dominance) ranged from 0.3 to 7.4% for the original phenotypic values (Fig. 1a and Table 3) and from 0.4 to 14.2% using the corrected phenotypic values (Fig. 1b and Table 4). The average increase in the observed prediction accuracies due to the use of haplotypes across all eight traits was 3.3% for the original phenotypic values and 3.2% for the corrected phenotypic values (Table 4). The detailed analysis of the prediction accuracies will focus first on results for the original phenotypic values, and then on the comparison of the results for the original and corrected phenotypic values.

**Table 3:** Accuracy of the best prediction models with haplotype additive values compared to the best SNP models

	Trait							
	AGW	ADG	BJS	FCR	LMA	LMD	BF	TN
SNP accuracy for predicting phenotypic values ${\hat{\text{R}}}_{{0}_{\text{jp}}}=\text{corr}({\hat{\text{g}}}_{{0}_{\text{j}}},{\text{y}}_{0})$								
A-only, Model-6	0.251	0.258	*0.258*	0.197	*0.402*	0.363	0.387	0.401
A + D, Model-5	*0.252*	*0.259*	0.244	*0.202*	0.401	*0.364*	*0.387*	*0.405*
A + D over A (%)	0.381	0.328	− 5.588	2.624	− 0.123	0.496	0.174	0.856
Best SNP prediction model (the SNP model in italic font, A-only or A + D)								
${ \hat{\text{R}}}_{{0}_{\text{jp}}}=\text{corr}({\hat{\text{g}}}_{{0}_{\text{j}}},{\text{y}}_{0})$	0.252 ± 0.032	0.259 ± 0.028	0.258 ± 0.033	0.202 ± 0.052	0.402 ± 0.042	0.364 ± 0.055	0.387 ± 0.061	0.405 ± 0.055
${\text{ R}}_{{0}_{\text{jp}}}={\text{R}}_{{0}_{\text{j}}}\sqrt{{\text{h}}_{\text{j}}^{2}}$	0.285 ± 0.010	0.290 ± 0.009	0.156 ± 0.013	0.241 ± 0.012	0.432 ± 0.008	0.418 ± 0.010	0.407 ± 0.015	0.405 ± 0.010
${\text{ R}}_{{0}_{\text{j}}}=\text{corr}({\hat{\text{g}}}_{{0}_{\text{j}}},{\text{g}}_{{0}_{\text{j}}})$	0.613 ± 0.010	0.624 ± 0.01	0.559 ± 0.016	0.579 ± 0.015	0.757 ± 0.004	0.722 ± 0.009	0.718 ± 0.010	0.707 ± 0.007
Haplotype prediction accuracy								
Best model	H	H	H	D + H	H	A + D + H	A + D + H	A + D + H
Best blocking	500 kb	500 kb	100 kb	1 Mb	Genes	Genes	1 Mb	Genes
${ \hat{\text{R}}}_{{0}_{\text{jp}}}=\text{corr}({\hat{\text{g}}}_{{0}_{\text{j}}},{\text{y}}_{0})$	0.270 ± 0.029	0.276 ± 0.027	0.277 ± 0.026	0.212 ± 0.066	0.413 ± 0.043	0.371 ± 0.055	0.392 ± 0.062	0.406 ± 0.053
Accuracy increase (%)	7.14	6.56	7.36	4.95	2.74	1.92	1.29	0.25
${\text{ R}}_{{0}_{\text{jp}}}={\text{R}}_{{0}_{\text{j}}}\sqrt{{\text{h}}_{\text{j}}^{2}}$	0.292 ± 0.006	0.298 ± 0.005	0.178 ± 0.011	0.248 ± 0.014	0.431 ± 0.006	0.417 ± 0.010	0.413 ± 0.015	0.401 ± 0.010
${\text{ R}}_{{0}_{\text{j}}}=\text{corr}({\hat{\text{g}}}_{{0}_{\text{j}}},{\text{g}}_{{0}_{\text{j}}})$	0.647 ± 0.005	0.650 ± 0.004	0.572 ± 0.012	0.549 ± 0.012	0.743 ± 0.004	0.710 ± 0.008	0.693 ± 0.009	0.695 ± 0.007

${\hat{\text{R}}}_{{0}_{\text{jp}}}$, observed accuracy of predicting phenotypic values; ${\text{R}}_{{0}_{\text{jp}}}$, theoretical accuracy of predicting phenotypic values; ${\text{R}}_{{0}_{\text{j}}}$, theoretical accuracy of predicting genotypic values; accuracy increase is the percentage increase in observed accuracy of predicting phenotypic values under the best haplotype model relative to the observed accuracy of the best SNP model (in italic font); A, SNP additive values; D, SNP dominance values; H, haplotype additive values; AGW, age at 100 kg live weight; ADG, daily gain; BJS, body judging score; FCR, feed conversion ratio; LMA, loin muscle area; LMD, loin muscle depth; BF, back fat thickness; TN, teat number

**Table 4:** Observed accuracy of predicting original and corrected phenotypic values for the best SNP and haplotype models

	Trait								
	AGW	ADG	BJS	FCR	LMA	LMD	BF	TN	Mean
Best SNP prediction model (as defined in Table 3)									
${ \hat{\text{R}}}{^{\text{s}}_{0\text{y}}}$	0.252	0.259	0.258	0.202	0.402	0.364	0.387	0.405	0.316
${\hat{\text{R}}}{^{\text{s}}_{0\text{r}}}$	0.256	0.262	0.138	0.204	0.415	0.394	0.399	0.407	0.309
Haplotype prediction accuracy									
Best model	H	H	H	D + H	H	A + D + H	A + D + H	A + D + H	
Best blocking	500 kb	500 kb	100 kb	1 Mb	Genes	Genes	1 Mb	Genes	
${ \hat{\text{R}}}{_{0\text{y}}^{\text{h}}}$	0.270	0.276	0.277	0.212	0.413	0.371	0.392	0.406	0.327
${\hat{\text{R}}}{_{0\text{r}}^{\text{h}}}$	0.269	0.274	0.157	0.211	0.426	0.400	0.403	0.409	0.319
${\hat{\text{R}}}{^{\text{s}}_{0\text{y}}}/{\hat{\text{R}}}{^{\text{s}}_{0\text{r}}}-1,\%$	− 1.0	0.5	88.9	− 1.5	0.3	− 2.9	− 1.7	− 3.6	
${\hat{\text{R}}}{^{\text{h}}_{0\text{y}}}/{\hat{\text{R}}}{^{\text{h}}_{0\text{r}}}-1,\%$	0.7	2.7	74.6	0.3	− 0.2	− 2.4	− 1.8	− 3.8	

${\hat{\text{R}}}{^{\text{s}}_{0\text{y}}}$, observed accuracy of predicting phenotypic values by the best SNP model using the original phenotypic values; ${\hat{\text{R}}}{^{\text{s}}_{0\text{r}}}$, observed accuracy of predicting phenotypic values by the best SNP model using the corrected phenotypic values; ${\hat{\text{R}}}{^{\text{h}}_{0\text{y}}}$, observed accuracy of predicting phenotypic values by the best haplotype model using the original phenotypic values; ${\hat{\text{R}}}{^{\text{h}}_{0\text{r}}}$, observed accuracy of predicting phenotypic values by the best haplotype model using the corrected phenotypic values; A, SNP additive values; D, SNP dominance values; H, haplotype additive values; AGW, age at 100 kg live weight; ADG, daily gain. BJS: body judging score; FCR, Feed conversion ratio; LMA, loin muscle area; LMD, loin muscle depth; BF, back fat thickness; TN, teat number

### Increased prediction accuracy with fixed-size haplotypes

**Fig. 2:**
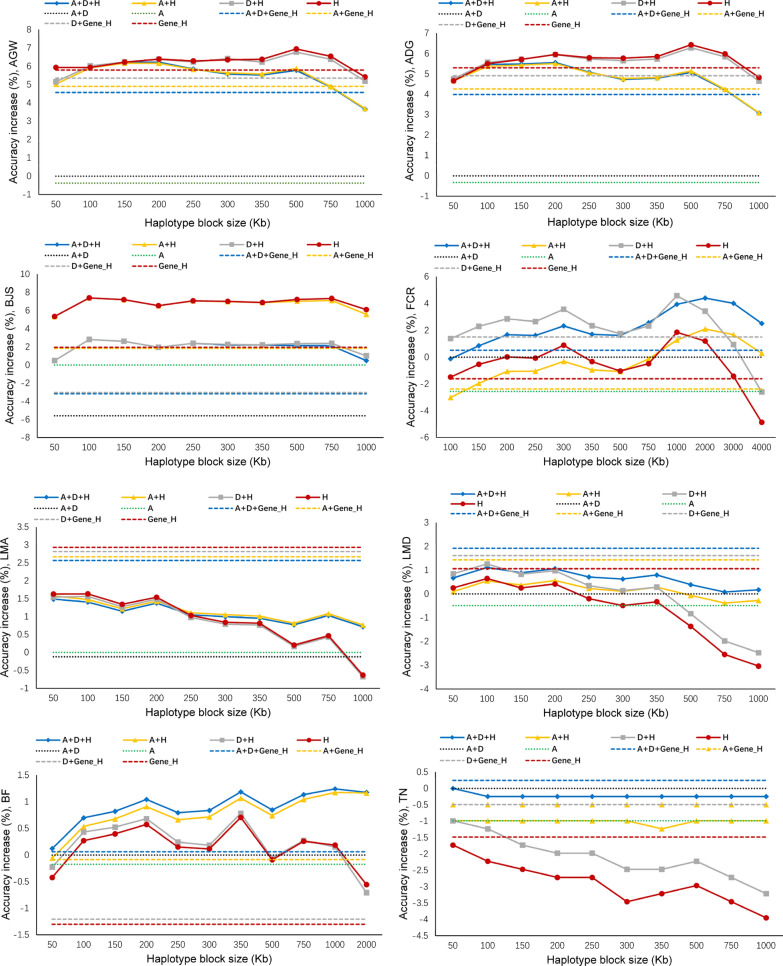
Observed prediction accuracy of haplotype models using fixed chromosome distance and gene boundaries per haplotype block. A = SNP additive values. D = SNP dominance values. H = haplotype additive values. Gene_H = gene-based haplotype additive values. *AGW* age at 100 kg live weight, *ADG* daily gain during, *BJS* body judging score, *FCR* Feed conversion ratio, *LMA* loin muscle area at 100 kg, *LMD* loin muscle depth at 100 kg, *BF* back fat thickness at 100 kg, *TN* teat number

For haplotype blocks defined by fixed chromosome distance, predictions of the original phenotypic values based on haplotype additive values (Model-4) had the highest accuracy for BJS, AGW and ADG, with increases in accuracy relative to the best SNP prediction of 7.4, 7.1 and 6.6% using haplotype block sizes of 100, 500, and 500 kb, respectively (Table 3). The full model (Model-1) with haplotype additive values of 1000-kb haplotype blocks and SNP additive and dominance values improved the prediction accuracy by 5.0% for FCR and 1.3% for BF (Table 3). The increase in prediction accuracy due to the use of haplotypes relative to the best SNP model was higher for AGW than for ADG, which was due to the lower SNP prediction accuracy of AGW, i.e., 0.252 for AGW and 0.259 for ADG (Table 3). For BJS, the high prediction accuracy due to the use of haplotypes was observed for all sizes of haplotype blocks evaluated, whereas 1- to 2-Mb haplotype blocks had the highest prediction accuracy for FCR, and 350 to 750-kb haplotype blocks had the highest prediction accuracy for AGW and ADG (Fig. 2). The error bars in Fig. 1 show that the traits with lower prediction accuracies (AGW, ADG, and BJS) had lower standard deviations of the observed prediction accuracies across validation populations than traits with higher prediction accuracies (LMA, LMD, BF and TN). The only exception was FCR, which had the lowest prediction accuracy but the largest standard deviation of observed prediction accuracies for unknown reasons.

### Increased prediction accuracy with gene-based haplotypes

Haplotype blocks defined by gene boundaries had the best prediction accuracy of original phenotypic values for two muscle traits (LMA and LMD) and teat number (TN), with increases in prediction accuracy of 2.7% for LMA, 1.9% for LMD and 0.3% for TN relative to the prediction accuracy of the best SNP model (Fig. 1 and Table 3). The haplotype-only model (Model-4) was the best prediction model for LMA, while the full model (Model-1) was best for LMD and TN. These results indicate that functional genomic information is relevant for haplotype genomic prediction and that haplotype prediction models could be an effective method to use functional genomic information (autosomal genes in this case) for genomic prediction of some traits. These results also provide examples showing whole-genome haplotype prediction is not always better than gene-based haplotype prediction, although the latter covered only 56.5% of the autosomes. A study on seven human traits reported that gene-based haplotype prediction was the best prediction model for one trait, although genes covered only 50.8% of all autosomes, and was tied for the best for the other traits [11]. These results provide evidence that the use of autosomal genes can result in the best haplotype prediction models for some quantitative traits.

### Comparison between observed and theoretical prediction accuracies

For the seven traits with increased prediction accuracies due to the use of haplotypes (except TN), the observed prediction accuracy [${\hat{\text{R}}}_{{0}_{\text{jp}}}$ of Eq. (7)] was lower than the theoretical prediction accuracy [${\text{R}}_{{0}_{\text{jp}}}$ of Eq. (10)] for predicting the original phenotypic values for six of the traits but was substantially higher than the theoretical accuracy for BJS under the best SNP and haplotype models. The ${\hat{\text{R}}}_{{0}_{\text{jp}}}$ for BJS was 0.258 for the best SNP model and 0.277 for the best haplotype model but the ${\text{R}}_{{0}_{\text{jp}}}$ of BJS was 0.156 for the best SNP model and 0.178 for the best haplotype model. The value of ${\text{R}}_{{0}_{\text{jp}}}$ from Eq. (10) decreased as heritability decreased. Thus, the main reason for the low ${\text{R}}_{{0}_{\text{jp}}}$ values was due to the low heritability estimates: 0.097 under the additive haplotype model and 0.124 under the $\text{A}+\text{D}$ SNP model (Table 5), the lowest among all traits. These results also demonstrate that the theoretical accuracy for predicting phenotypic values was not always higher than the observed accuracy. The theoretical accuracy for predicting genetic values [${\text{R}}_{{0}_{\text{j}}}$ of Eq. (9)] was higher than the observed and theoretical accuracies for predicting phenotypic values for all haplotype and SNP models, as expected, because ${\text{R}}_{{0}_{\text{j}}}$ is the upper limit of ${\text{R}}_{{0}_{\text{jp}}}$. The comparison between the best haplotype and SNP models showed that the theoretical accuracy for predicting phenotypic values (${\text{R}}_{{0}_{\text{jp}}}$) under the best haplotype models was higher than under the best SNP models for five traits (AGW, ADG, BJS, FCR, and BF) and was lower for two traits (LMA and LMD), and the theoretical accuracy for predicting genetic values (${\text{R}}_{{0}_{\text{j}}}$) under the best haplotype models was higher than under the best SNP models for three traits (AGW, ADG, and BJS) and was lower for four traits (FCR, LMA, LMD, and BF). The higher haplotype ${\text{R}}_{{0}_{\text{jp}}}$ values for AGW, ADG, BJS, FCR, and BF were consistent with the higher haplotype heritability estimates than the SNP heritability estimates (Table 5), but the reason for the lower ${\text{R}}_{{0}_{\text{j}}}$ for FCR, LMA, LMD, and BF under the haplotype models than under the SNP models was unknown.

**Table 5:** Relationship between haplotype heritability and prediction accuracy for eight traits under the best prediction models

	Trait							
	AGW	ADG	BJS	FCR	LMA	LMD	BF	**TN**
SNP model with additive values (A)								
Additive heritability ${(\hat{\text{h}}}{_{{\upalpha }1}^{2}})$	0.182	0.186	*0.078*	0.142	*0.327*	0.315	0.300	0.297
SNP model with additive and dominance values (A + D)								
Additive heritability ${(\hat{\text{h}}}{_{{\upalpha }2}^{2}})$	0.173	0.179	0.076	0.139	0.327	0.309	0.299	0.293
Dominance heritability ${(\hat{\text{h}}}{_{\updelta }^{2}})$	0.045	0.038	0.048	0.036	0.000	0.027	0.028	0.037
SNP broad-sense heritability ${(\hat{\text{h}}}{_{\text{s}}^{2}})$	*0.218*	*0.216*	0.124	*0.175*	0.327	*0.336*	*0.326*	*0.331*
Haplotype prediction models								
Best model	H	H	H	D + H	H	A + D + H	A + D + H	A + D + H
Best haplotype blocking method	500 Kb	500 Kb	100 Kb	1 Mb	Genes	Genes	1 Mb	Genes
Accuracy increase (%)	7.14	6.56	7.36	4.95	2.74	1.92	1.29	0.25
SNP additive heritability ${(\hat{\text{h}}}{_{{\upalpha} {\text{s}}}^{2}})$	–	–	–	–	–	0.101	0.154	0.137
SNP dominance heritability ${(\hat{\text{h}}}{_{{\updelta} {\text{s}}}^{2}})$	–	–	–	0.036	–	0.026	0.022	0.037
Haplotype additive heritability ${(\hat{\text{h}}}{_{{\upalpha} {\text{h}}}^{2}})$	0.208	0.213	0.097	0.170	0.336	0.215	0.183	0.162
Total heritability ${(\hat{\text{h}}}{_{\text{g}}^{2}})$	0.208	0.213	0.097	0.206	0.336	0.343	0.359	0.336
Estimates of haplotype epistasis heritability								
Haplotype epistasis heritability ${(\hat{\text{h}}}{_{\text{g}}^{2}})$	0.026	0.027	0.019	0.031	0.009	0.007	0.033	0.005
Relative haplotype epistasis heritability ${(\hat{\text{h}}}{_{\text{Er}}^{2}}, \%)$	14.29	14.52	24.36	22.30	2.75	2.26	11.04	1.71

Accuracy increase is the percentage increase in observed prediction accuracy of the best haplotype model relative to the accuracy of the best SNP model (in italic font) using the original phenotypic values

*A* SNP additive values, *D* SNP dominance values, *H* haplotype additive values, *AGW* age at 100 kg live weight, *ADG* daily gain, *BJS* body judging score, *FCR* feed conversion ratio, *LMA* loin muscle area, *LMD* loin muscle depth, *BF* back fat thickness, *TN* teat number

### Comparison between observed accuracies for predicting the original and corrected phenotypic values

The observed accuracies for predicting the original and corrected phenotypic values were two observed correlations: the correlation between the GBLUP of genotypic values and the original phenotypic values, and the correlation between the GBLUP of genotypic values and the corrected phenotypic values after removing the fixed year-season effects in each validation population. The results of these observed accuracies showed that the haplotype models had better prediction accuracies than the SNP models for all traits for predicting the original and corrected phenotypic values (Fig. 1) and that, on average across the eight traits, the SNP and haplotype prediction accuracies for the original and corrected phenotypic values were similar. Two fixed effect levels did not have observations in the training populations and these two observations were removed when calculating the observed prediction accuracies using the corrected phenotypic values. Increases in accuracy when including haplotypes were greater for the original phenotypic values than for the corrected phenotypic values for AGW, ADG and FCR, with increases in accuracy of 7.1, 6.6 and 5.0%, respectively, for the original phenotypic values (Fig. 1a and Table 3), and of 4.7, 4.6 and 3.4% for the corrected phenotypic values (Fig. 1b and Table 4). Removing fixed effects resulted in minor changes of the increases in accuracy from including haplotypes for LMA, LMD, BF and TN, but in a substantial increase for BJS, i.e., an increase in accuracy of 14.2% using the corrected phenotypic values. For the same SNP model, corrected phenotypic values had a higher prediction accuracy for five traits (AGW, FCR, LMD, BF and TN) and a lower prediction accuracy for three traits (ADG, BJS and LMA). For the same haplotype model, corrected phenotypic values had a higher prediction accuracy for four traits (LMA, LMD, BF and TN) and a lower prediction accuracy for four traits (AGW, ADG, BJS and FCR). The reason for the large decrease in the observed prediction accuracy due to the removal of fixed effects for BJS was unclear: 88.9% lower for the SNP model and 74.6% lower for the haplotype model compared to prediction of the original phenotypic values (Table 4). These BJS results should indicate the presence of inconsistency for grading the BJS scores at certain time periods. On average across the eight traits, predictions were more accurate for the original phenotypic values than for the corrected phenotypic values for both the SNP and haplotype models. The average observed prediction accuracy under the SNP models was 0.316 for the original and 0.309 for the corrected phenotypic values, and the average observed prediction accuracy under the haplotype models was 0.327 for the original and 0.319 for the corrected phenotypic values (Table 4).

### SNP additive and dominance heritabilities and impacts on prediction accuracy

Estimates of the SNP additive heritability ranged from 0.08 (for BJS and FCR) to 0.33 (for LMA), while estimates of SNP dominance heritability ranged from 0.00 (for LMA) to 0.05 (for BJS) (Table 5). The inclusion of dominance effects increased the prediction accuracy by 2.5% for FCR, which had a dominance heritability of 0.04, but decreased prediction accuracy by 5.7% for BJS, which had the highest dominance heritability (0.05). Inclusion of SNP dominance effects slightly decreased the prediction accuracy for LMA (− 0.3%)*,* slightly increased the prediction accuracy for AGW (0.4%), AGD (0.4%), LMD (0.3%), and TN (1.0%), and had no effect on the prediction accuracy for BF (Table 3).

### Haplotype epistasis heritability and impact on prediction accuracy

**Fig. 3:**
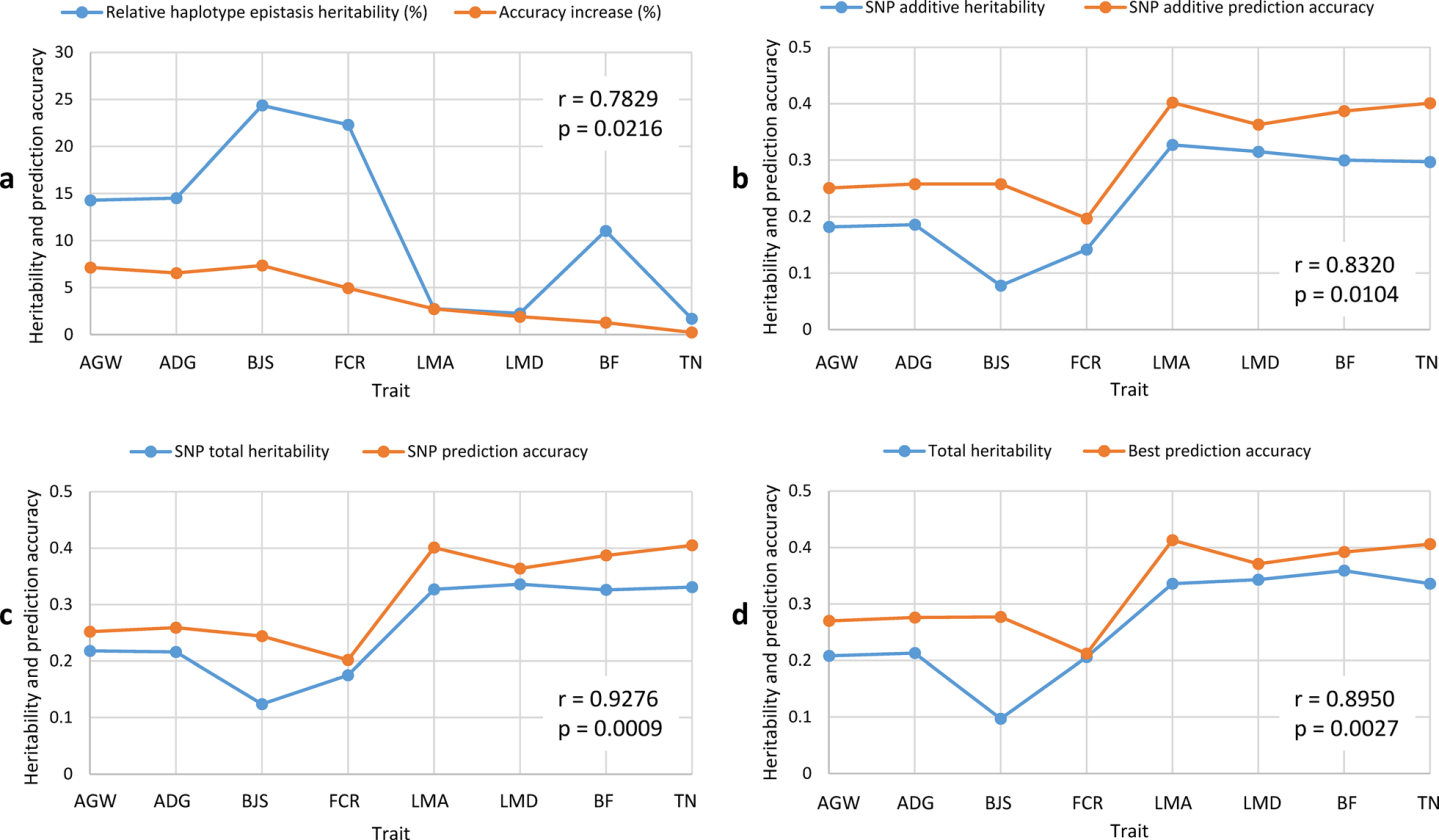
Relationship between observed prediction accuracy and heritability estimates. **a** Correlation between relative increase in prediction accuracy due to haplotypes and relative haplotype epistasis heritability. **b** Correlation between prediction accuracy of the best haplotype model and total heritability that can be haplotype heritability only or a combination of haplotype and SNP heritabilities. **c** Correlation between prediction accuracy of the SNP model with additive and dominance values and the SNP total heritability as a summation of additive and dominance heritabilities. **d** Correlation between prediction accuracy of the SNP model with additive values and SNP additive heritability. *AGW* age at 100 kg live weight, *ADG* daily gain duringv *BJS* body judging score, *FCR* Feed conversion ratio, *LMA* loin muscle area at 100 kg, *LMD* loin muscle depth at 100 kg, *BF* back fat thickness at 100 kg, *TN* teat number

The relationship between heritability estimates and prediction accuracy is the basis to understand the performance of different prediction models. We examined the relationship between estimates of relative haplotype epistasis heritability [Eqs. (21) and (22)] and the increase in prediction accuracy due to the use of haplotypes (Table 5). The four traits (BJS, AGW, ADG, and FCR) with the highest relative haplotype epistasis heritability estimates (14.3 to 24.4%) also had the largest increases in haplotype prediction accuracy (5.0 to 7.4%). The three traits (LMA, LMD, and TN) with the lowest relative haplotype epistasis heritability estimates (1.7 to 2.8%) had three of the four smallest increases in haplotype prediction accuracy (0.3 to 2.7%). The correlation between estimates of relative haplotype epistasis heritability and the increase in accuracy due to the use of haplotypes was statistically significant (r = 0.78, p = 0.02, Fig. 3a). These results were in strong agreement with the results on human data [11], i.e., haplotype epistasis was mainly responsible for the increased accuracy of haplotype prediction models given that haplotype epistasis was the only new genetic information generated by haplotypes and that the relative haplotype heritability was strongly correlated with the increase in prediction accuracy. As a comparison, the correlation was also significant between estimates of SNP additive heritability and prediction accuracy (r = 0.83, p = 0.01, Fig. 3b), between estimates of SNP total heritability and SNP prediction accuracy (r = 0.93, p = 0.0009, Fig. 3c), and between estimates of total heritability of the best prediction model and the best prediction accuracy (r = 0.90, p = 0.003, Fig. 3d). These comparisons showed that the correlation between estimates of relative haplotype epistasis heritability and accuracy increase due to the use of haplotypes (Fig. 3a) had a similar statistical significance but was not as significant as the correlations between prediction accuracy and estimates of the three types of heritability (Fig. 3b–d). In a human haplotype genomic prediction study, the correlation between estimates of relative haplotype epistasis heritability and the increase in prediction accuracy due to the use of haplotypes was more significant than the other three correlations [11]. These results of high correlations between relative haplotype epistasis heritability and accuracy increase for swine and human data showed that haplotype epistasis was mainly responsible for the increase in prediction accuracy of haplotype genomic prediction.

### Comparison of heritability profiles of SNPs and haplotypes

The differences between heritability profiles across the genome based on SNPs and haplotypes reflect the differences between the SNP and haplotype models in the genetic contributions of genes and chromosome regions to phenotypic variation. Such differences in heritability profiles provide indications about the likely reason why a haplotype model does or does not improve prediction accuracy. Our results showed that the haplotype heritability profile needs to be different from the SNP heritability profile, at least for some regions, for the haplotype models to be more accurate than the SNP models. For traits where the haplotype-only models were most accurate, haplotype effects fully accounted for the SNP effects because adding SNPs to the prediction model decreased the prediction accuracy. In these cases, the chromosomal locations with high heritability estimates should be considered as more accurately identified than those with high SNP heritability estimates but are not shared by haplotypes. For traits where the integration of SNP and haplotype additive values increased the prediction accuracy over the haplotype-only models, haplotypes likely incorrectly estimated some SNP effects and the inclusion of SNPs in the prediction model compensated the weakness of haplotypes in those cases. One trait (TN) provided an example where SNP and haplotype heritability profiles were virtually identical, and the use of haplotypes virtually provided no help for improving prediction accuracy (increased prediction accuracy by only 0.25%).

#### Heritability profiles for AGW, ADG, BJS and FCR

**Fig. 4:**
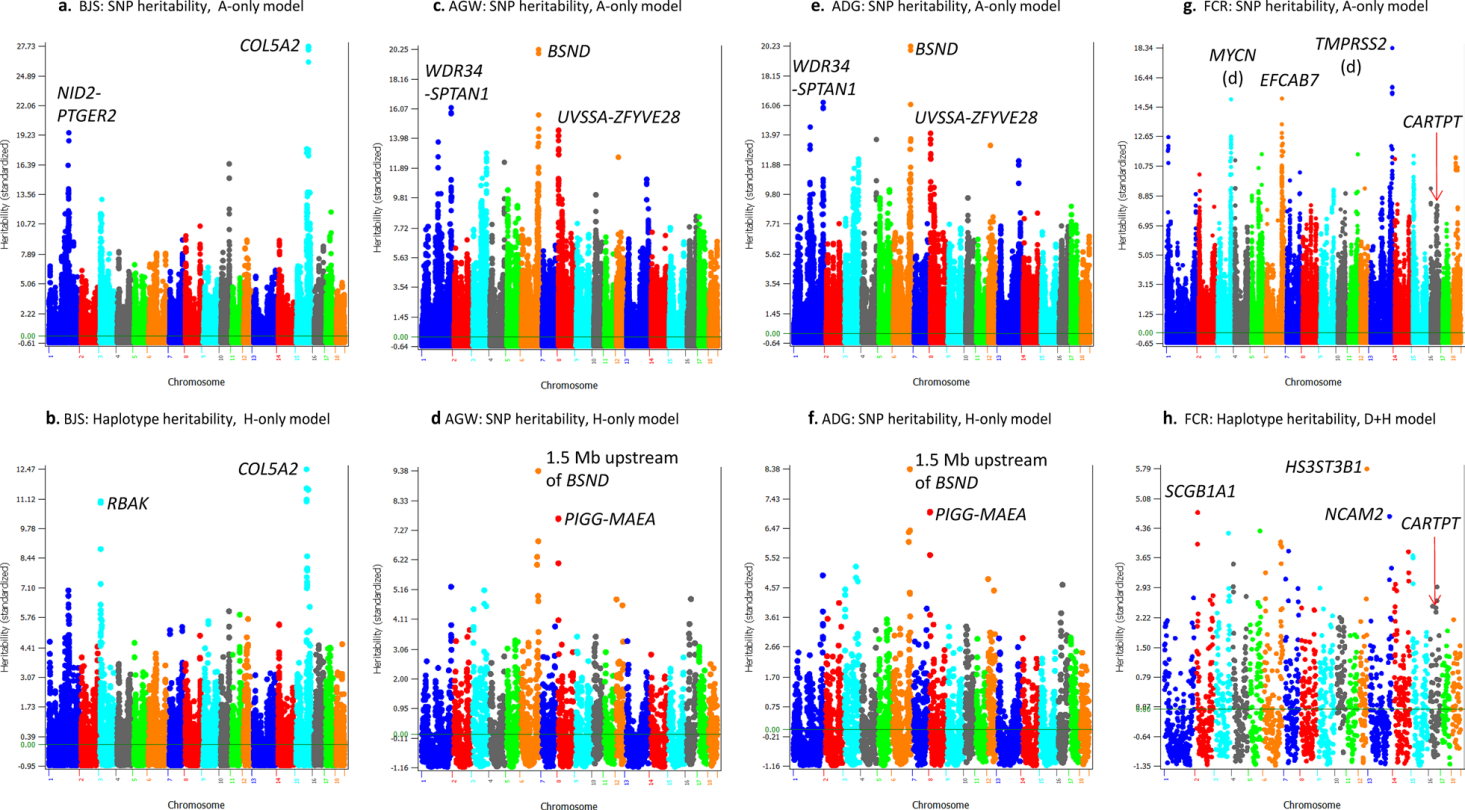
Heritability profiles of SNPs and haplotypes for body judging score (BJS), age at 100 kg live weight (AGW), average daily gain (ADG), and feed conversion ratio (FCR). **a** SNP heritability profile of BJS; **b** Haplotype heritability profile of BJS; **c** SNP heritability profile AGW; **d** Haplotype heritability profile of ADG; **e** SNP heritability profile of average daily gain ADG; **f** Haplotype heritability profile of ADG; **g** SNP heritability profile of FCR; and **h** Haplotype heritability profile of FCR

The haplotype-only model (Model-4) had the best prediction accuracy for AGW, ADG, BJS and LMA, and the $\text{D}+\text{H}$ model (Model-3) had the best prediction accuracy for FCR. A common feature of these models is the absence of SNP additive values. The SNP and haplotype heritability profiles identified common and different regions with high heritability estimates. Chromosome locations with a high haplotype heritability should be more accurately identified than those with high SNP heritability estimates because of the higher prediction accuracy of the haplotype-only models over the SNP models. For BJS, both SNP and haplotype heritability profiles identified the *COL5A2* gene as having the highest heritability (Fig. 4a and b), but the second highest heritability estimate for BJS was for the region that included the *NID2-PTGER2* genes by SNP analysis (Fig. 4a) and for the *RBAK* gene by haplotype analysis (Fig. 4b). For AGW, the SNP heritability profile identified the *BSND* gene region as having the highest heritability **(**Fig. 4c), but the haplotype heritability profile identified the chromosome region that is 1.5-Mb upstream of *BSND* as having the highest heritability **(**Fig. 4d). AGW (Fig. 4c and d) virtually had identical SNP and haplotype heritability profiles as ADG (Fig. 4e and f), providing confirmation that AGW and ADG were associated with the same genetic factors. The largest differences or least overlap between the highest SNP and haplotype heritability profiles were observed for FCR (Fig. 4g and h). FCR had the highest SNP additive heritability estimate for the region that included the *TMPRSS2* gene on chromosome 13 (Fig. 4g) but had the highest haplotype heritability estimate for the *HS3ST3B1* gene on chromosome 12 (Fig. 4h). Since SNP additive values were not in the prediction model, the haplotype heritability estimates should have fully accounted for the SNP heritability estimates for FCR. The *CARTPT* gene, also known as *CART*, is involved in the regulation of appetite and energy homeostasis [33]. For FCR, the *CARTPT* gene did not have the highest haplotype heritability, but still had high haplotype heritability estimates (Fig. 4h), and a haplotype block immediately downstream of *CARTPT* had the same haplotype heritability estimate as that in the haplotype block that included *CARTPT*. Therefore, *CARTPT* likely has a substantial contribution to the phenotypic variance of FCR based on the haplotype heritability estimates. The SNP heritability estimates also indicated a substantial contribution of *CARTPT* to FCR, because the total SNP heritability of the 20 SNPs in the 48.02–48.07 Mb region on chromosome 16, which contains the *CARTPT* gene, was slightly higher than the sum of the heritability estimates of all 11 SNPs in the 204.91–204.95 Mb region on chromosome 13, which had the highest SNP heritability estimates among all SNPs (Fig. 4g).

#### Heritability profiles of muscle and fat traits

**Fig. 5:**
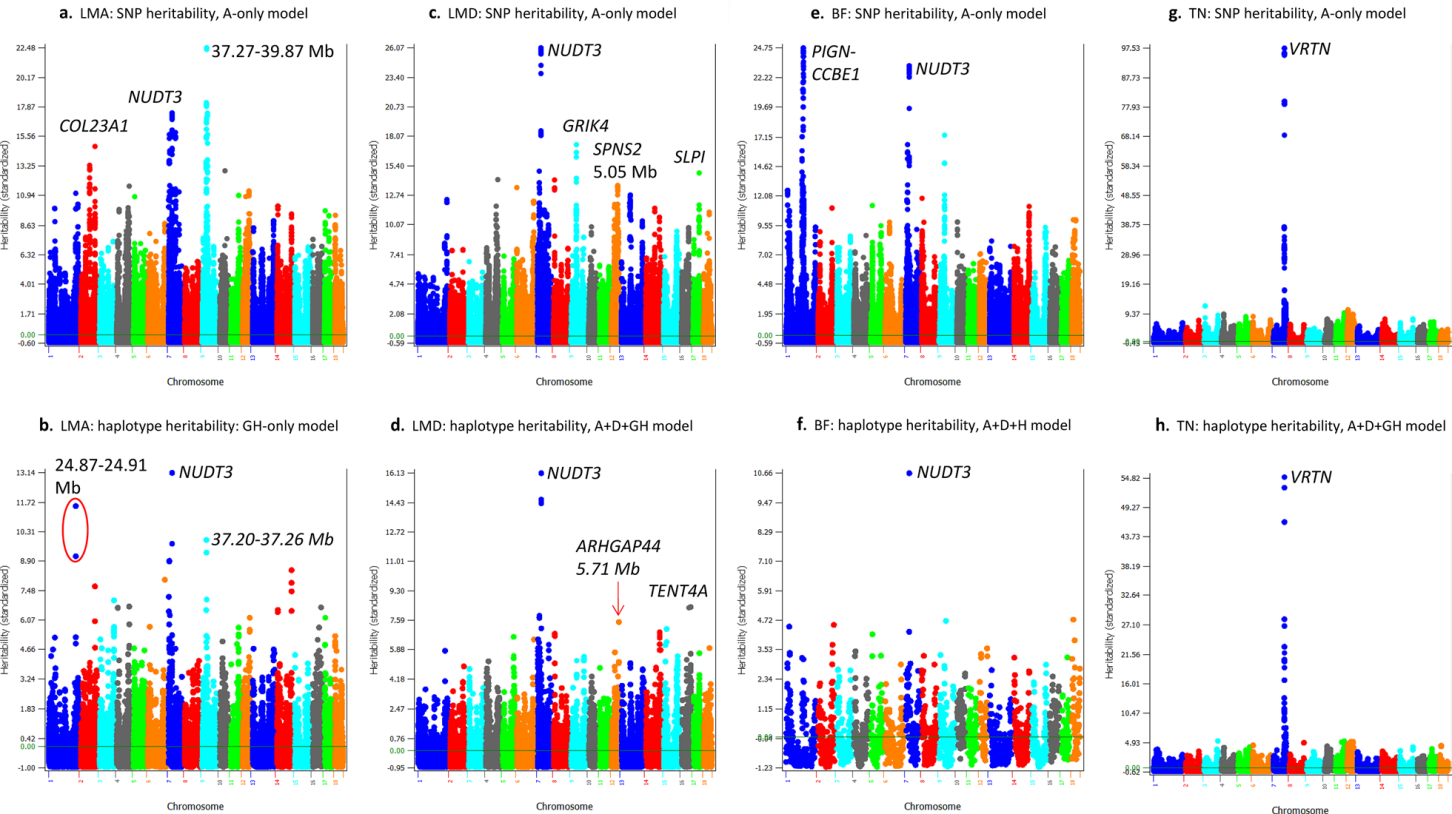
Heritability profiles of SNPs and haplotypes for loin muscle area (LMA), loin muscle depth (LMD), backfat (BF), and teat number (TN). **a** SNP heritability profile of LMA; **b** Haplotype heritability profile of LMA; **c** SNP heritability profile of LMD; **d** Haplotype heritability profile of LMD; **e** SNP heritability profile of BF; **f** Haplotype heritability profile of BF; **g** SNP heritability profile of TN; **h** Haplotype heritability profile of TN

The heritability profiles of SNPs and haplotypes for the three muscle and fat traits, LMA, LMD, and BF, all identified the *NUDT3* gene as having high SNP and haplotype heritability estimates (Fig. 5). For LMA, the best prediction model was the haplotype-only model. SNP heritability profiles identified *NUDT3* as having the second highest SNP heritability (Fig. 5a) but the haplotype heritability profiles identified this gene as having the highest haplotype heritability (Fig. 5b). This result was expected since the haplotype model had a higher prediction accuracy. The reasons for the increase in prediction accuracy due to the use of haplotypes for LMA included a more accurate estimate of the sum of small effects by haplotypes than the estimate of each small effect by SNPs, noting that LMA had one of the smallest haplotype epistasis heritability estimates, explaining less than 1.0% of the phenotypic variance (Table 5). The integration of SNP and haplotype additive values resulted in the best prediction model for four traits including FCR discussed in the previous section, LMD, BF, and TN. For LMD, *NUDT3* had the highest SNP and haplotype heritability estimates (Fig. 5c and d), *GRIK4* had the second highest SNP heritability estimate (Fig. 5c), and *TENT4A* had the second highest haplotype heritability estimate (Fig. 5d). For BF, *NUDT3* had the second highest SNP heritability and the highest haplotype heritability estimates (Fig. 5e and f), and *PIGN-CCBE1* had the second highest SNP heritability estimate (Fig. 5e), but the haplotype model identified six locations with similar haplotype heritability estimates, on chromosomes 1, 2, 5, 7, 9 and 18, that were much lower than the haplotype heritability for *NUDT* (Fig. 5f). It is interesting to note that the *NUDT3* gene had high SNP and haplotype heritability estimates for all three muscle and fat traits (LMA, LMD, and BF), which was consistent with previous results that NUDT3 had significant effects for LMA and LMD [34] and for BF [15] in Duroc pigs. The accuracy increases due to the integration of SNPs with haplotypes indicated that haplotypes alone did not capture all the SNP information for these traits, a phenomenon termed as ‘haplotype loss’ [32], which was compensated by including SNPs in the prediction model. Given the accuracy increases due to SNPs, the haplotype loss for FCR, LMD and BF was due to less accurate or insufficient estimation of SNP effects. For FCR, dominance effects were unaccounted for by haplotype additive effects. For LMD, the *GRIK4* with large SNP heritability estimates did not have high haplotype heritability estimates, and for BF, *PIGN-CCBE1* with large SNP heritability estimates did not have high haplotype heritability estimates. These differences in SNP and haplotype heritability profiles likely contributed to the increased prediction accuracy due to the integration of SNPs with haplotypes and this integration compensated the haplotype loss for those traits. The next example was the only known example showing virtually identical SNP and haplotype heritability profiles with no accuracy increase from the haplotype model over the SNP model.

#### Heritability profiles for TN

The SNP and haplotype heritability profiles for TN were unique because they were virtually identical, with high SNP and haplotype heritability estimates within and around the *VRTN* gene on chromosome 7 (Fig. 5g and h). Such an absence of differences in heritability profiles is probably due to the absence of haplotype epistasis, and it should be noted that TN had the smallest relative haplotype epistasis heritability (1.7%, Table 5). TN was the only example for which the haplotype analysis did not improve prediction accuracy when profiles of SNP and haplotype heritability estimates were virtually identical. Several reports confirmed that the *VRTN* gene and its surrounding regions had the most significant effects on TN [24, 35–38]. Although the heritability estimates for this region were about 10 times as high as the highest estimates for other regions, this region only accounted for 10.0% of the genomic additive heritability and 8.0% of the observed accuracy of genomic prediction [24]. Therefore, the discussion on the high heritability obtained for TN and for the other traits is to compare heritability profiles under different models and does not deny the relevance of chromosome regions with low heritability estimates to the accuracy of genomic prediction.

## Conclusions

Analysis of haplotype genomic prediction models showed that haplotype prediction models had a higher prediction accuracy of phenotypic values than SNP models in Duroc pigs. Overall, the traits analyzed in this study had different SNP and haplotype heritability profiles and required different haplotype prediction models to achieve the best prediction accuracy. Haplotype-only models were the best prediction models for some traits, whereas the integration of SNP and haplotype effects in the prediction model provided the best prediction accuracy for other traits. Gene-based haplotype blocks resulted in the best prediction accuracy for some traits, providing evidence that gene-based haplotypes contained the most important genetic information for those traits although they only covered part of the autosomes.

## Supplementary Information

**Additional file 1: Table S1.** Trait statistics of the Duroc population. This table provides basic statistics for the eight traits analyzed in the Duroc population used in this study. **Figure S1.** Phenotypic distributions. AGW: age at 100 kg live weight. ADG: daily gain during 30–100 kg live weight. FCR: Feed conversion ratio during 30–100 kg. LMA: loin muscle area at 100 kg. LMD: loin muscle depth at 100 kg. BF: back fat thickness at 100 kg. TN: teat number. BJS: body judging score. “FCR without outliers” means phenotypic values that were more than four standard deviations from the mean were removed. **Figure S2.** Heat map of the SNP density distribution across the autosomes. This figure provides a global view of the SNP coverage of the swine autosomes in the Duroc population used in this study. **Figure S3.** Distribution of autosomal gene sizes. This figure shows the distribution of the gene sizes on swine autosomes.

## Data Availability

The data sets supporting the results of this article are included within the article and its additional files. The phenotypic data and original SNP data are private property of Guangdong Wens Foodstuffs Group and are not currently available for public distribution.
