# Supplementary material for: Haplotype genomic prediction of phenotypic values based on chromosome distance and gene boundaries using low-coverage sequencing in Duroc pigs
Source: Genet Sel Evol. 2021 Oct 7;53:78. doi: 10.1186/s12711-021-00661-y (PMC8496108; doi:10.1186/s12711-021-00661-y)
Supplement: Supplementary file 1 — Additional file 1: Table S1. Trait statistics of the Duroc population. This table provides basic statistics for the eight traits analyzed in the Duroc population used in this study. Figure S1. Phenotypic distributions. AGW: age at 100 kg live weight. ADG: daily gain during 30–100 kg live weight. FCR: Feed conversion ratio during 30–100 kg. LMA: loin muscle area at 100 kg. LMD: loin muscle depth at 100 kg. BF: back fat thickness at 100 kg. TN: teat number. BJS: body judging score. “FCR without outliers” means phenotypic values that were more than four standard deviations from the mean were removed. Figure S2. Heat map of the SNP density distribution across the autosomes. This figure provides a global view of the SNP coverage of the swine autosomes in the Duroc population used in this study. Figure S3. Distribution of autosomal gene sizes. This figure shows the distribution of the gene sizes on swine autosomes. [file 12711_2021_661_MOESM1_ESM.pdf]

**Table S1 Trait statistics of the Duroc study population**

| Trait | N | Mean | SD | Min | Max |
|---|---|---|---|---|---|
| AGW | 3194 | 154.82 | 9.32 | 124.47 | 203.01 |
| ADG | 3193 | 636.67 | 37.89 | 483.72 | 781.71 |
| BJS | 3194 | 8.23 | 0.47 | 6 | 10 |
| FCR | 3041 | 2.17 | 0.20 | 1.27 | 3.04 |
| LMA | 3193 | 36.32 | 3.57 | 24.19 | 51.04 |
| LMD | 3194 | 46.65 | 4.08 | 33.9 | 63.4 |
| BF | 3189 | 10.91 | 2.1 | 6.01 | 19.84 |
| TN | 3195 | 10.73 | 1.07 | 8 | 15 |

AGW: age to 100 kg live weight. ADG: daily gain at 100 kg live weight. BF: back fat thickness at 100 kg. LMA: loin muscle area at 100 kg. LMD: loin muscle depth at 100 kg. TN: teat number. SD: standard deviation. Min: minimum value. Max: maximum value.

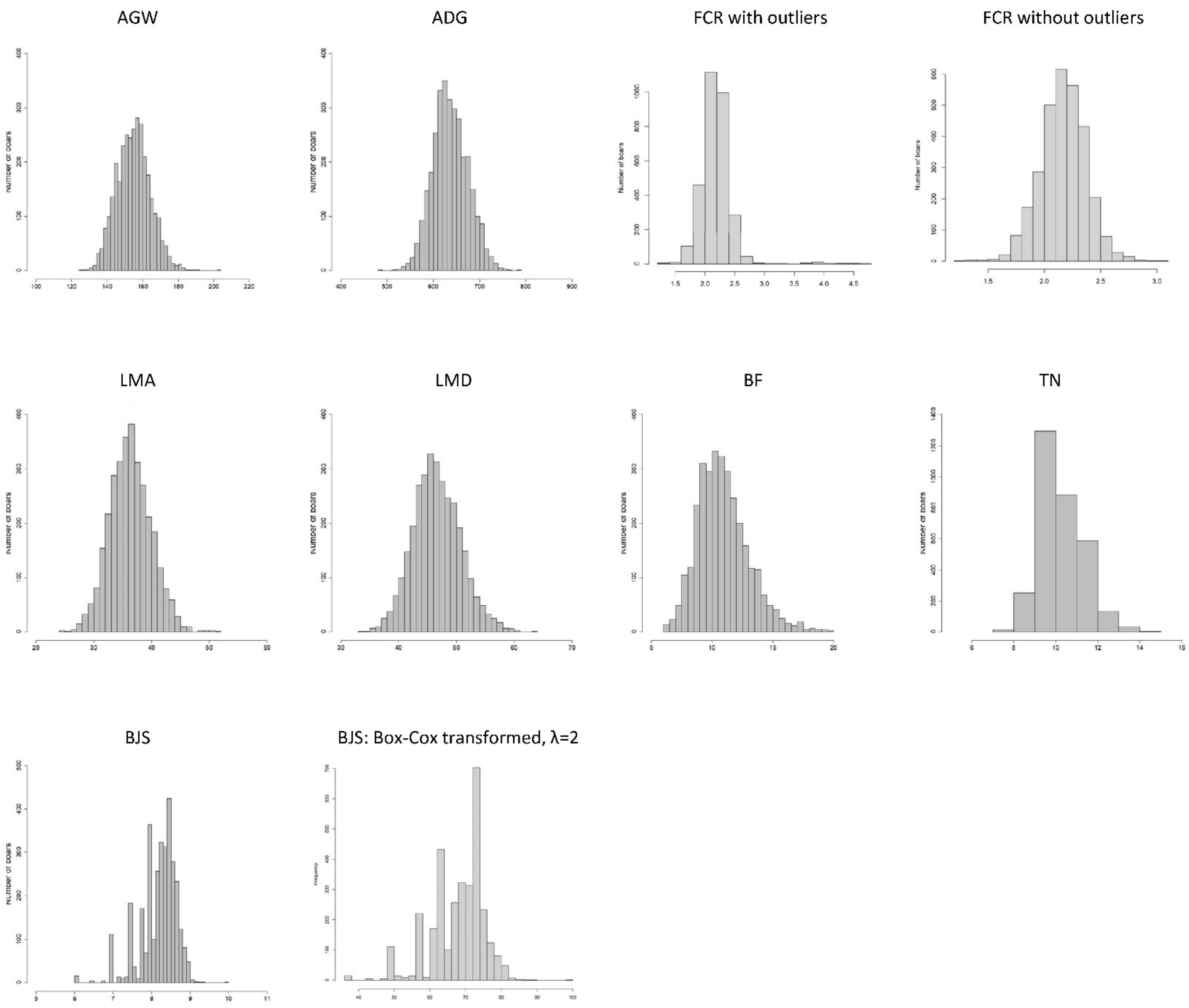


**Figure S1.** Phenotypic distributions. AGW: age at 100 kg live weight. ADG: daily gain during 30-100 kg live weight. FCR: Feed conversion ratio during 30-100 kg. LMA: loin muscle area at 100 kg. LMD: loin muscle depth at 100 kg. BF: back fat thickness at 100 kg. TN: teat number. BJS: body judging score. “FCR without outliers” means phenotypic values that were more than four standard deviations from the mean were removed.

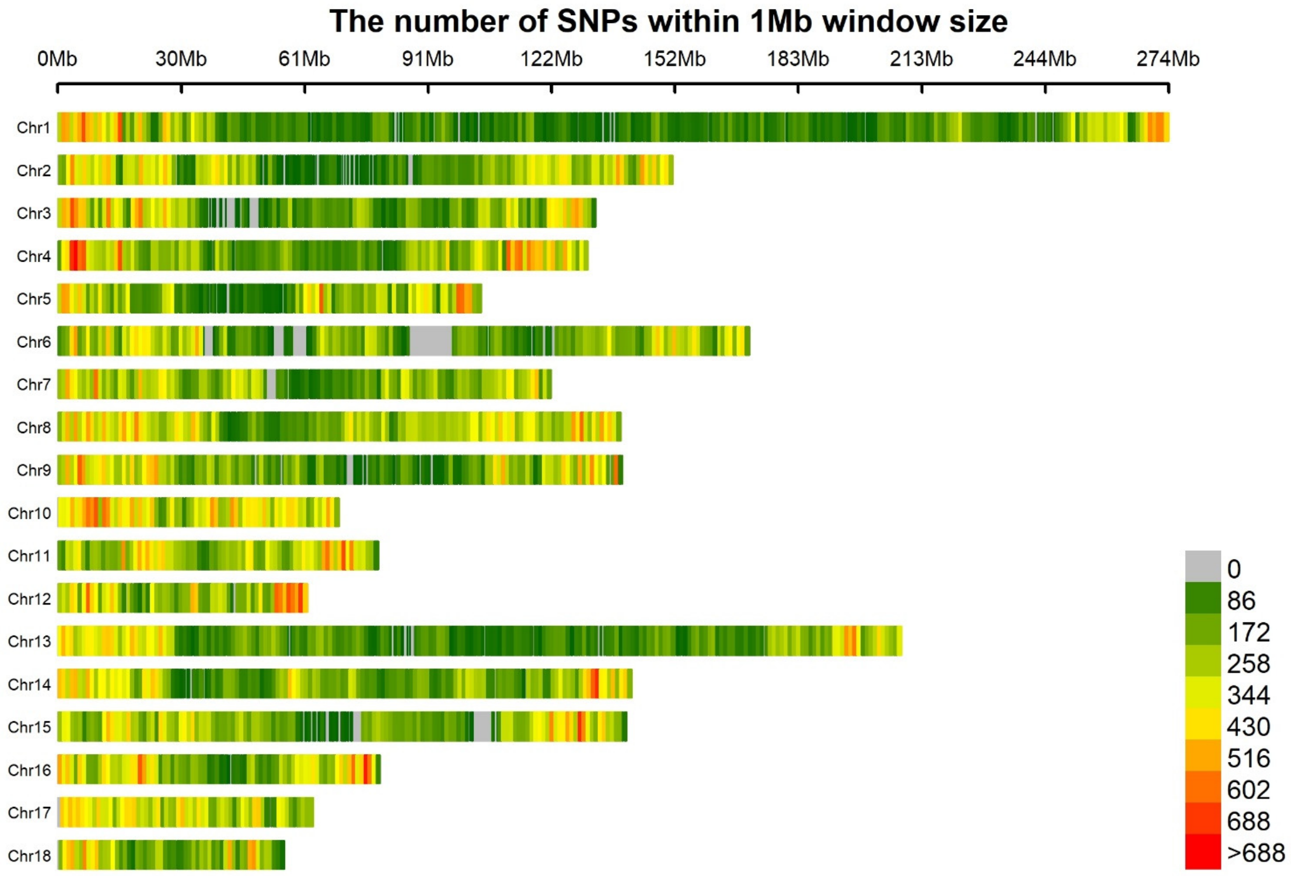


**Figure S2.** Heat map of the SNP density distribution across the autosomes. This figure provides a global view of the SNP coverage of the swine autosomes in the Duroc population used in this study.

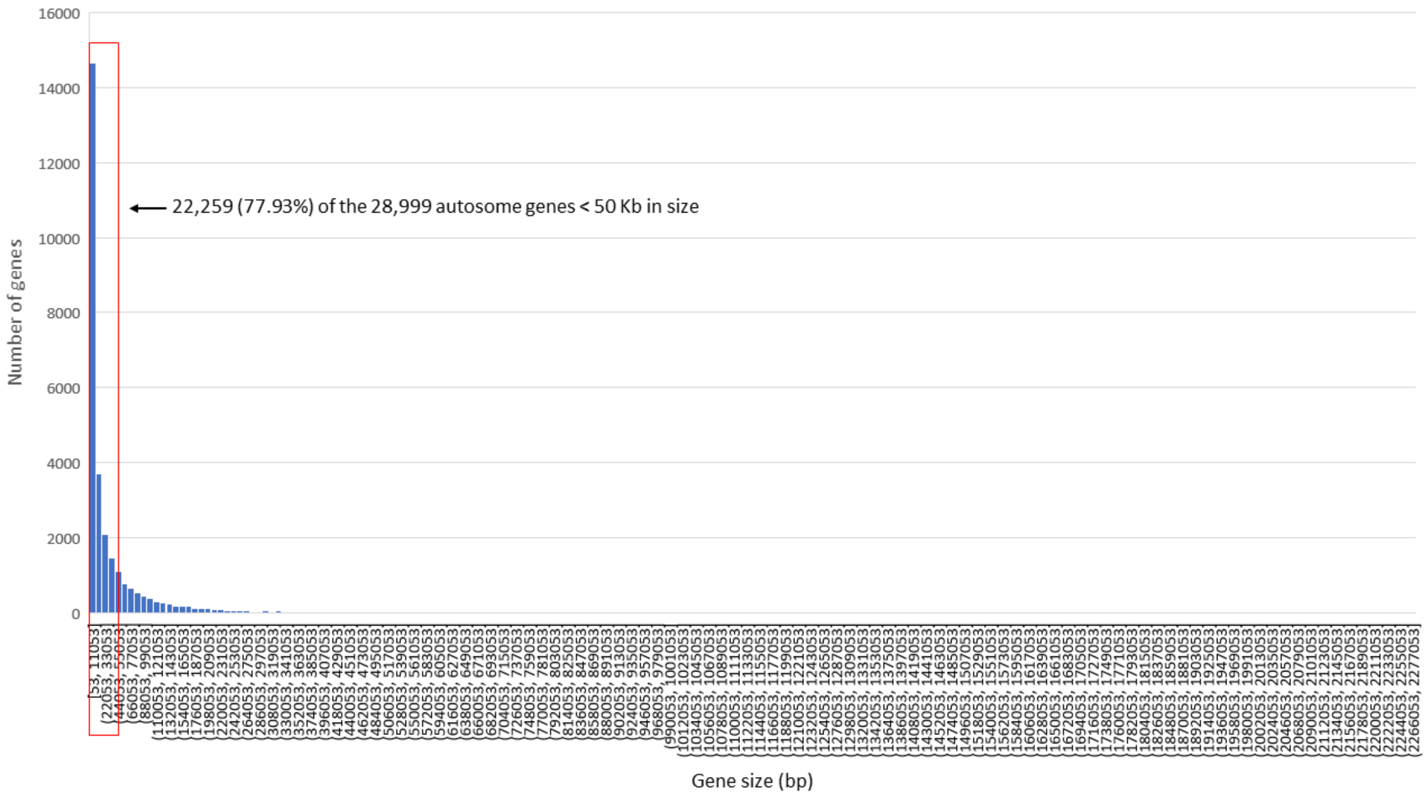


**Figure S3.** Distribution of autosomal gene sizes. **T**his figure shows the distribution of the gene sizes on swine autosomes.
